# Human Body-Related Disease Diagnosis Systems Using CMOS Image Sensors: A Systematic Review

**DOI:** 10.3390/s21062098

**Published:** 2021-03-17

**Authors:** Suparshya Babu Sukhavasi, Susrutha Babu Sukhavasi, Khaled Elleithy, Shakour Abuzneid, Abdelrahman Elleithy

**Affiliations:** 1 Department of Computer Science and Engineering, University of Bridgeport, Bridgeport, CT 06604, USA; susukhav@my.bridgeport.edu (S.B.S.); ssukhava@my.bridgeport.edu (S.B.S.); abuzneid@bridgeport.edu (S.A.); 2 Department of Computer Science, William Paterson University, Wayne, NJ 07470, USA; elleithya@wpunj.edu

**Keywords:** CMOS, CMOS image sensors, medical imaging systems, medical applications, biomedical CMOS image sensors, implantable CMOS image sensors, smartphone CMOS image sensors

## Abstract

According to the Center for Disease Control and Prevention (CDC), the average human life expectancy is 78.8 years. Specifically, 3.2 million deaths are reported yearly due to heart disease, cancer, Alzheimer’s disease, diabetes, and COVID-19. Diagnosing the disease is mandatory in the current way of living to avoid unfortunate deaths and maintain average life expectancy. CMOS image sensor (CIS) became a prominent technology in assisting the monitoring and clinical diagnosis devices to treat diseases in the medical domain. To address the significance of CMOS image ‘sensors’ usage in disease diagnosis systems, this paper focuses on the CIS incorporated disease diagnosis systems related to vital organs of the human body like the heart, lungs, brain, eyes, intestines, bones, skin, blood, and bacteria cells causing diseases. This literature survey’s main objective is to evaluate the ‘systems’ capabilities and highlight the most potent ones with advantages, disadvantages, and accuracy, that are used in disease diagnosis. This systematic review used PRISMA workflow for study selection methodology, and the parameter-based evaluation is performed on disease diagnosis systems related to the human body’s organs. The corresponding CIS models used in systems are mapped organ-wise, and the data collected over the last decade are tabulated.

## 1. Introduction

**Figure 1:**
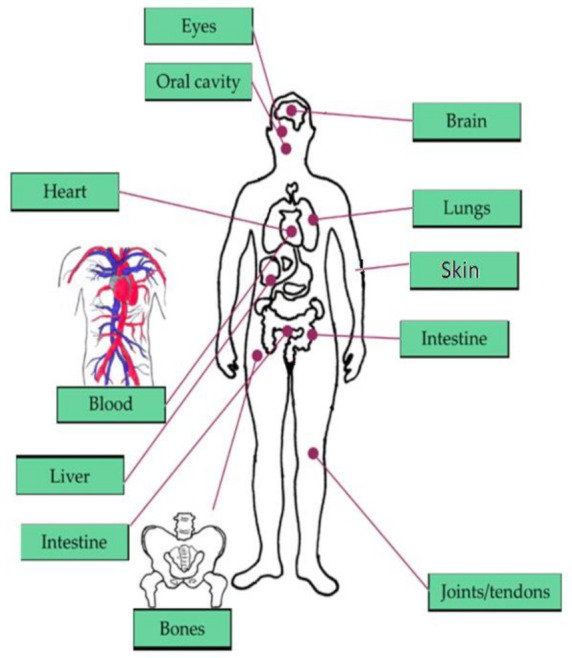
Vital signs and organs of the human body focused in our literature survey [9].

The brain, heart, eyes, intestines, and lungs are the most affected organs in the current living way. The monitoring of human organs like the brain, eyes, heart, intestine, lungs, and vital signs (shown in Figure 1) observation is mandatory for secure and healthy human living to avoid early deaths. According to the year 2019 statistics [1], the death rate caused by heart failure is 647,000 every year, and death is recording for every 37 s as per CDC because of cardiovascular disease. The heart’s major diseases are cardiac arrest, coronary artery disease (CAD), congestive heart failure, aortic disease, peripheral arterial disease, etc. According to a global burden disease study [2] conducted on neurological disorders from 1990 to 2017, around 100 million Americans are being affected by one or more neurological disorders. Among them, Alzheimer’s, Parkinson’s, migraine, and stroke are the foremost neurological disorders leading to death. The average death rate of Alzheimer’s disease is 258,600 deaths, Parkinson’s disease is 30,000 deaths, and stroke is 172,000 deaths. According to Leukemia & Lymphoma Society [3], a death is recording due to blood cancer for every nine minutes, six deaths are recording for every hour, 156 deaths are recording for every day, and the estimated death rate in the United States would be 56,840 by the end of 2020 due to leukemia, lymphoma, and myeloma which are the major blood disorders. In addition, diabetes [4] is becoming the seventh major cause of death in the United States by recording 270,702 deaths in 2017. By the year 2040 [5], an estimation of 78.4 million people over the age introduction of 18 years or older will affect by arthritis, of which two-thirds of them would be women. For every five Americans, one person is developing skin cancers during their lifetime, leading to an estimation of 9500 every day [6]. The major skin cancers are melanoma, non-melanoma skin cancer, basal cell carcinoma, and squamous cell carcinoma, affecting 3 million people every year. By the year 2020, 6850 deaths are recorded due to melanoma skin cancer. By 2050 [7], it is estimated that 8.96 million people will become either visually impaired or blind as per CDC. The national institute of occupational safety and health report says 2000 USA workers are prone to eye accidents every day. According to CDC [8], 14.8 million adults are diagnosed with cancers, in which 266,000 people are diagnosed with gastro intestine (GI) cancers, and 14,400 deaths are recorded due to GI cancers in 2014.

In our survey, we concentrate on literature related to the CMOS image sensors assisting medical devices in diagnosing diseases affecting vital signs and parts of the human body. Our contributions are listed below:

- We have conducted a novel systematic review on CIS utilization in disease diagnosis in the medical field.
- We have extracted data and evaluated by specifying the vital parameters required for medical systems performing disease diagnosis shown in Table 2.
- Based on our literature survey, we have tabulated all the available technical specifications related to CMOS image sensors in Appendix A Table A1.

The remainder of the paper is organized as follows. We used PRISMA workflow for study selection methodology in Section 2. In Section 3, we discussed CIS’s role in diagnosing the diseases affecting the human body and the data extraction with evaluation in Section 4. Organ wise and year wise utilization of CIS models in disease diagnosis systems are discussed in Section 5. In Section 6, conclusions are offered.

## 2. Study Selection Methodology

**Figure 2:**
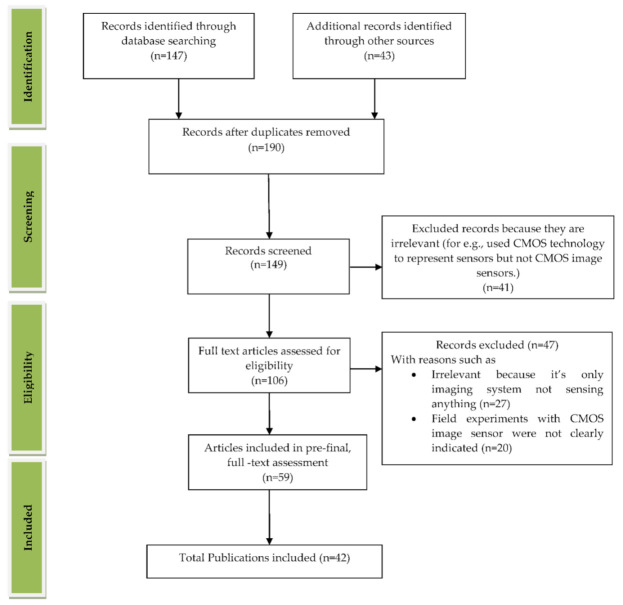
PRISMA workflow for the process of article selection. Figure adapted with permission from [10].

**Figure 3:**
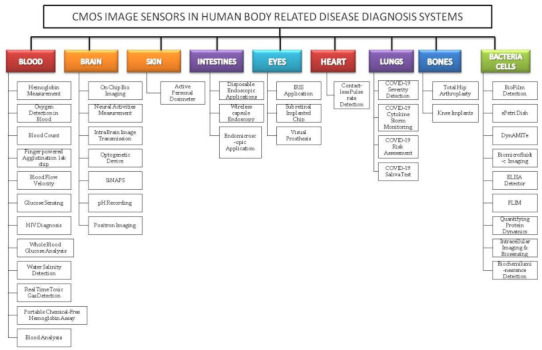
Classification of complementary metal-oxide semiconductor image sensor (CIS) embedded disease diagnosis systems relating to vital signs and organs of the human body.

We reviewed the literature covering the years between 2009 and 2021 that is drawn from the article search engines like “Google Scholar”, “IEEE Xplore”, “SpringerLink”, “MDPI”, “Pub Med”, “ELSEVIER”, “ARXIV”, “Scopus”, “Science citation index (SCI)”, “SPIE Digital Library”, “ACM Digital Library”, “ASME.” The review was conducted systematically by a PRISMA workflow, as shown in Figure 2. Different tools were available for searching and selection of publications like “DOAJ,” “EndNote,” and “Microsoft Excel.” We included the publications involving CMOS image sensors used in medical applications to monitor different body parts like the brain, bones, eyes, blood, intestine, heart, and lungs. We classified the searched publications into qualitative, quantitative, book, and editorial categories. The entire search was conducted based upon the words used in the “keyword” section. We searched and collected articles of about 190 papers, and finally, we bound the selection to 42 full-text articles from the year 2009 to the year 2021. All the 42 papers are cited in the reference section, and “EndNote” software was used for managing the references. As our literature survey is conducted on CMOS image sensors in the medical field, we primarily focused on the articles from “Elsevier” and “Google Scholar.” We excluded the articles that are not in English, particularly those irrelevant since the imaging systems used were not sensing anything. Some articles are also excluded because they did not mention the usage of CIS in their field experiments. The other articles excluded are duplicate articles, unreachable articles. The entire flowchart for the selection process, including identification, screening, eligibility, and inclusion, is shown in Figure 2 [10]. Based on the study selection methodology using PRISMA workflow, we have classified our literature survey according to vital signs and organs of the human body-related disease diagnosis systems embedded with CMOS image sensors, as shown in Figure 3.

## 3. Role of CIS in Human Body-Related Disease Diagnosis Systems

An image sensor is a sensor that receives the incident photons from light and converts them into electrons. Two types of image sensors are introduced into the market; charge-coupled device (CCD) and CMOS image sensors based on complementary metal-oxide semiconductor (CMOS) technology. Due to the expensive manufacturing process and specific fabrication, and high-power consumption of CCD’s, CMOS image sensors are preferred in most of the fields in current-day technology. There are two types of pixel structures present in CMOS image sensors, namely, passive pixel sensors and active pixel sensors. Passive pixel sensors are introduced earlier with no amplification inside the circuitry, and because of column capacitance, high noise and low sensitivity is occurring in passive pixel sensors. However, active pixel sensors lead over the passive pixel sensors due to its advantages of amplifier incorporated into the pixel. Increased pixel performance and power consumption are also very less compared to CCD’s. CMOS image sensors are playing a prominent role in medical sciences in such a way that they are implemented in every part of the body. Around 15 million deaths were caused by ischemic heart disease and stroke globally, and finding the methods to identify them nowadays is crucial. Diagnostic modules like X-ray, magnetic resonance imaging (MRI), computer tomography, and echocardiography are used to rescue many lives. Still, these modules can produce black and white images only. However, the colored images can provide a lot of information to the physicians to detect and apply suitable treatment. The improvements in CIS technology bring more quality color images. Its miniature size can incorporate anywhere into the human body for diagnosis and provide the patient information accurately to the physicians for better treatments to save lives.

CIS’s involvement in biomedical applications is rapidly increasing day by day, and technical advancements are being made to meet the design specifications of present-day human needs, due to CIS’s compatibility in terms of its characteristics that are high dynamic range, less power consumption, low manufacturing cost, and on-chip functionality. These characteristics made CIS the preferred imaging component in most biomedical applications to perform all its functions alone or with less power.

Because of the high costs in the health care system, the time duration in hospital stays is getting shorter day by day, and patients are returning home with sickness and need a continuous health monitoring process. The patients with chronic diseases should be monitored continuously, especially the old aged population, increasing every year, preferring to live alone and do not want to be in an assisted living facility. Sophisticated technology makes the monitoring devices be minimized with built-in battery or portable power sources leading to a novel, innovative world of possibilities. For instance, cell phones that are portable and battery-operated can do real-time monitoring and control a lot of applications. Implantable and wearable technologies sense the parameters of different diseases. They will transfer the patient’s data to the treating facility or directly to the patient to take the corresponding action. For example, if the blood glucose levels cross the limits, this technology alerts the patient to take the required insulin amount. Before any implantable application be introduced into human society, sufficient trials will be conducted on rats, so here, we focused our survey in rat-based experiments to know the advancements in implantable applications that are not introduced into the market. Smartphones are becoming dependable devices in daily human living, especially in using smart home appliances, cars, and navigation. These smartphones are being used in medical fields as detectors, analyzers, and conducting diagnostic tests.

### 3.1. Disease Diagnosis Systems Related to Blood

**Figure 4:**
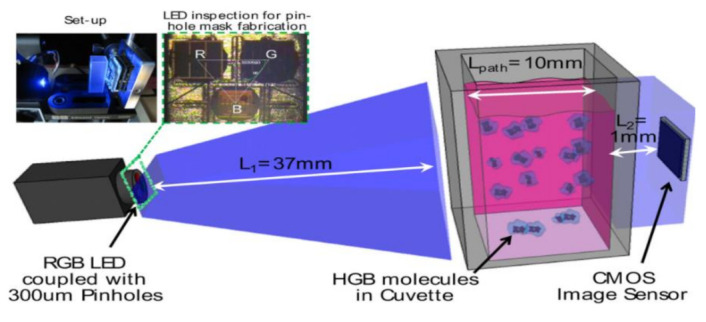
Experimental setup for hemoglobin concentration measurement using CIS. Figure adapted with permission from [11].

Quantifying hemoglobin concentration is an essential process in most clinical laboratories to analyze the postoperative bleeding, status of autologous blood transfusion, anemia, etc. Over 400 million hemoglobin tests for concentration are conducted every year to identify blood-related diseases and disorders in the United States. Dong-Sik Kim et al. [11] developed a new technique to make hemoglobin concentration measurement cost effective and straightforwardly. It provides accurate hemoglobin values from samples of blood without involving any reagents harmful to the environment and works with the help of LED and CIS as shown in Figure 4.

**Figure 5:**
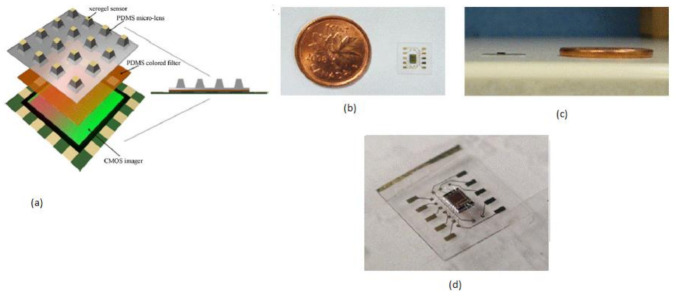
(**a**) Imaging sensor block diagram. (**b**) Ultra-thin packaged CIS with a Canadian one-cent coin as a reference. (**c**) Cross-sectional view. (**d**) A packed imager IC. Figure adapted with permission from [12].

Mini chemical sensors play a crucial role in medical applications like patient bed monitoring and disposal sample answer systems, personal safety, and implantable chips. Daisy S. Daivasagaya et al. [12] focused on luminescence sensors microarrays, which provide more advantages like a fast response, no additional reagents required, and will not spoil the sample media. He described a portable optical gaseous oxygen sensor microsystem using xerogel sensor elements, contact printed on the top of a trapezoidal lens like microstructures molded into polydimethylsiloxane (PDMS). PDMS is a soft, biocompatible, flexible, and an optically transparent silicon-organic polymer suitable to fabricate the lens, diffusers, and filters of the optical sensors. The imaging sensor system’s block diagram is shown in Figure 5.

**Figure 6:**
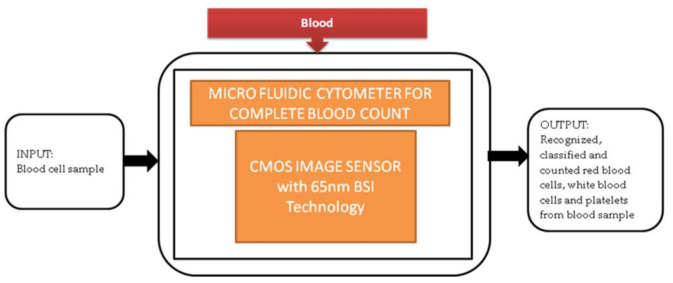
Microfluidic cytometer for a complete blood count.

Generally, in old-aged people, a complete blood count (CBC) is one of the resources for blood tests to find the health status of patients with heart diseases. Most of the CBC instruments like hematology analyzers and hemocytometers are implemented traditionally. These instruments need heavy equipment like some actuators, countertop, and liquid systems. So, these instruments are limited to laboratories in hospitals and research centers. An alternative solution to calculate CBC is using Raman scattering and optical microscopy. Xu Liu et al. [13] developed a super-resolution microfluidic cytometer to perform CBC tests. The microfluidic cytometer prototype consists of a PDMS microfluidic channel with attached CIS, PCB (printed circuit board) incorporated in FPGA board, and a MATLAB GUI working laptop shown in the paper. Its black-box approach is shown in Figure 6. This lens-free microfluidic cytometer is well suited for high accuracy whole blood recognition and its count in the point-of-care diagnosis of old-aged people.

**Figure 7:**
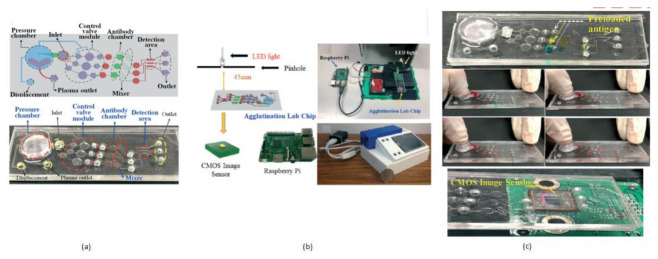
(**a**) Fingered powered agglutination lab chip. (**b**) Homemade CIS-based mini sensing system and finger powered agglutination lab chip (**c**) Antibodies were preloaded and pressing procedures, the agglutination lab chip is placed on CMOS image sensor of the homemade mini system. Figure adapted with permission from [14].

The term agglutination is an antigen and antibody reaction, which will happen during the clumping of visible particles formation. In this process, antibody combines with the respective antigen with the electrolytes at a specific pH and temperature. Chung Hsiang Lu et al. [14] developed a finger-powered agglutination lab chip with a CMOS image sensor outside that was affordable and cost-effective. For demonstration, blood grouping is used to verify the function of the finger power agglutination lab chip. The antibodies of blood are loaded before into the antibody reaction chamber in the lab chip. Then, the sample of blood will be pushed into the antibody reaction chamber by a finger-pressed power actuation to start the hemagglutination reaction to find the type of blood in the detection area of the on-chip CIS mini sensing system. Without external power, finger-based power actuation is well suitable for low-cost applications. This system will separate the red blood samples from the whole blood by pressing finger powered pump. The comprehensive blood test can be completed by pressing the finger 5–6 times on the agglutination-chip, as shown in Figure 7.

**Figure 8:**
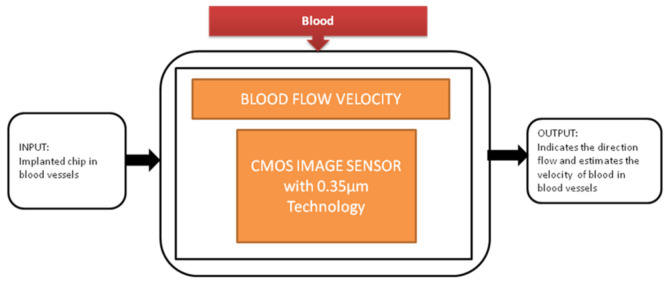
Blood flow velocity detection.

During the study of brain imaging, experiments will be conducted on small animals like rats under anesthesia because the brain’s activities will be hard to monitor under nonanesthetized conditions. Makito Haruta et al. [15] developed an ultra-small CMOS imaging device to monitor brain activities under non-anesthetized conditions. In addition, the author demonstrated the blood flow velocity detection in the brain using the CIS over a long period of non-anesthetized conditions. This imaging device is implanted in a rat head for demonstration shown in the paper cited. Its black-box approach is shown in Figure 8.

The future expectations of this device will contribute to the actual brain functional mechanisms of animals’ behavior.

**Figure 9:**
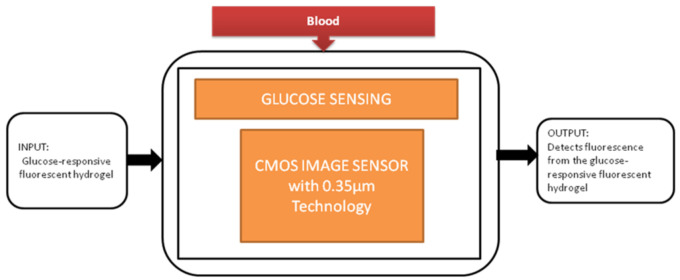
Implantable glucose sensing system.

The most familiar metabolic disease globally is diabetes, and its main symptom is the high level of glucose in the blood. It causes the obligations like kidney failure and loss of sight, so keeping the glucose levels under control every day is crucial for diabetic patients. To perform this glucose monitoring daily, a blood sample needs to be taken from the fingertip. Takashi Tokuda et al. [16] uses an optical sensing scheme, a unique technique in which glucose sensors use fluorescent hydrogel, and its black-box approach is shown in Figure 9.

**Figure 10:**
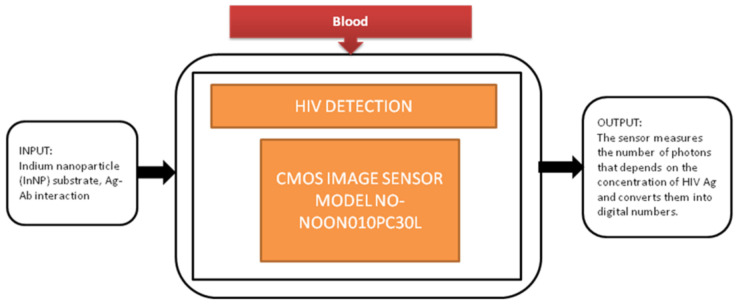
CMOS image sensor-based human immunodeficiency virus (HIV) detection.

Jasmine Pramila Devadhasan et al. [17] developed a CIS-based immunodiagnosis system to detect the (HIV) human immunodeficiency virus. The image sensor in the system counts the photons with respect to antigen concentration of HIV and converts the photon count into digital numbers. Its black-box approach is shown in Figure 10.

**Figure 11:**
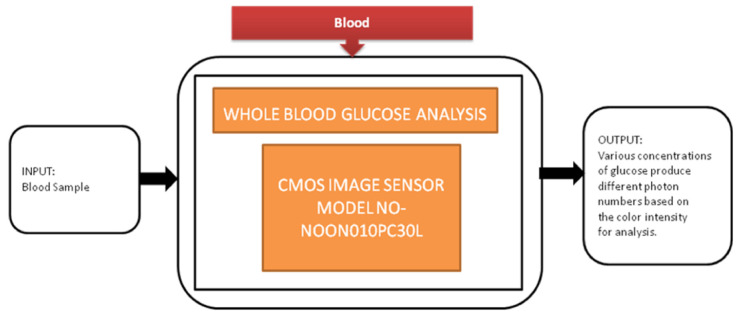
Whole glucose blood analysis.

Diabetes is the most common disease, which is increasing rapidly across the world. Regular monitoring the glucose levels is the most suitable method to control diabetes in old-aged people by not making them prone to peripheral neuropathy. Jasmine Pramila Devadhasam et al. [18] developed a whole blood glucose analysis system using a smartphone camera using a point-of-care methodology. Its black-box approach is shown in Figure 11.

Using the enzyme kinetic method, a glucose assay is taken, is reacted with the immobilized assay reagent, and then exposed to light that produces a color captured by CIS. The corresponding digital value is displayed on the smartphone screen.

**Figure 12:**
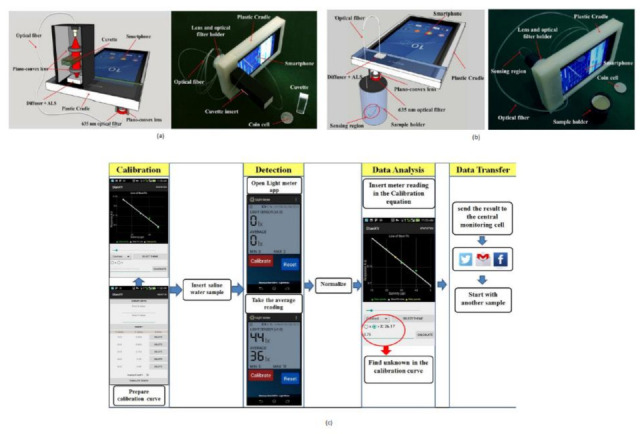
(**a**) Salinity detection using direct transmission smartphone-based approach. (**b**) Smartphone-based salinity detection using evanescent field absorption approach. (**c**) Steps involved in measuring salinity in water. Figure adapted with permission from [19].

Salinity detection is also one of the crucial parameters to monitor for the protection of ocean management. If the sea salt level is above the normal value, it may cause oxygen and osmosis concentration changes. Iftak Hussain et al. [19] developed a smartphone-based salinity sensor to monitor the sea environment’s salt level. Two types of methods are proposed for sensing based on direct transmission, and evanescent field absorption approaches are shown in Figure 12a,b.

The salinity detection and analysis are performed on two different Android platforms. Using a smartphone, one can communicate and share the data in real-time from the remote center to the seawater monitoring unit. The author also mentioned that this device is for measuring the daily salt intake measurement for diabetic patients.

**Figure 13:**
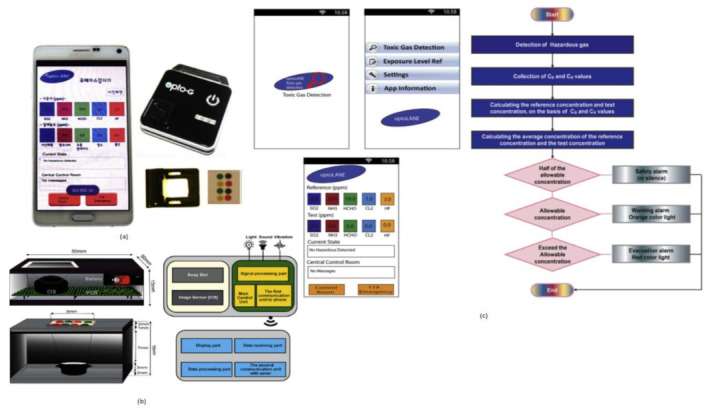
(**a**) Smartphone-based toxic gas detection system. (**b**) Instrumental setup for toxic gas detection microarray reader. (**c**) Stepwise illustration of smartphone application “Toxic Gas Detection.” Figure adapted with permission from [20].

Toxic gases like hydrogen fluoride, ammonia, and chlorine cause harmful effects on human beings like skin diseases and diarrhea and sometimes lead to death. Jasmine Pramila Devadhasan et al. [20] developed a smartphone-based toxic gas detection, which is a handheld operated device in real time. In this detection process, toxic gases will be detected with the help of titanium nanoparticles, which are blended with polyvinyl alcohol hydrogel strips then mixed with chemically reactive colors. These colors will change with respect to base acid reactions. The array strips will be monitored by a colorimetry system and sort them in chrominance data form. These signals will be sent to the smartphone and display the levels of toxic gases detected on the screen by opening the smartphone application called “toxic gas detection,” shown in Figure 13. The toxic gas detection also helps avoid exposure to different toxic gases, causing anemia, a blood-related disorder.

**Figure 14:**
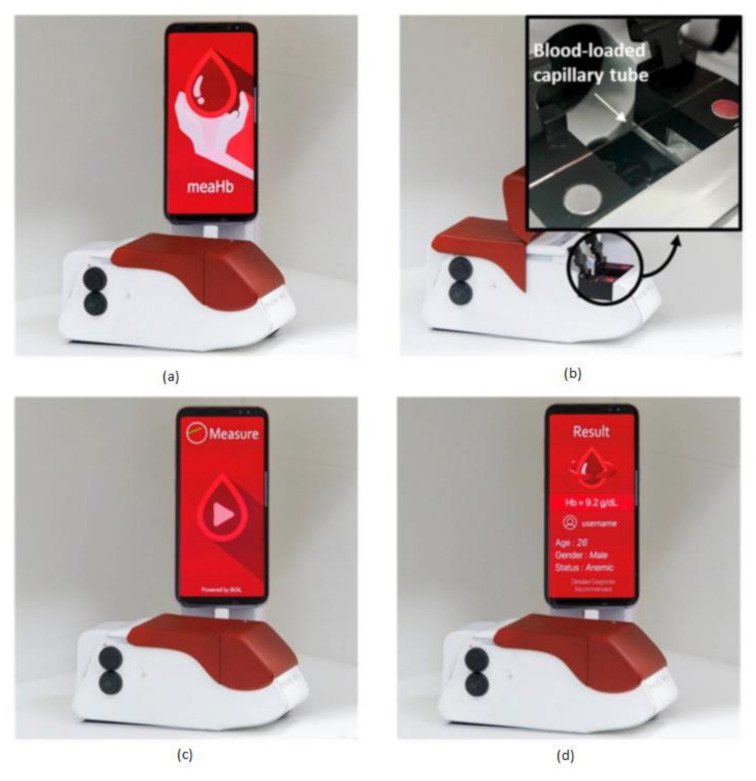
(**a**) Photo Thermal Angular Scattering technology (m-PTAS) sensor. (**b**) Blood sample capillary tube loaded into m-PTAS module. (**c**,**d**) Smartphone application “meaHb” displays the computed hemoglobin [21].

Anemia is a common blood disorder across the world and is also called as Global Burden of Disease. Particularly an iron-deficiency anemia, which suffers around 1.5 billion human lives, caused due to the insufficient amount of iron makes your body not produce enough red blood cells to carry the oxygen (hemoglobin). Hemoglobin deficiency causes dental disorders, neurological disorders, heart diseases, metabolic changes, and endocrine disorders [3]. Junho Lee et al. [21] developed a chemical-free smartphone-based hemoglobin concentration detection system using Photo Thermal Angular Scattering technology (PTAS) called in short m-PTAS, as shown in Figure 14.

This module is portable, chemical-free, and a speedy hemoglobin detection mechanism. Blood samples are taken into a capillary tube and loaded into m-PTAS module for computing and hemoglobin analysis. A dedicated android application called “meaHb” is developed to perform the hemoglobin analysis. This module is affordable, disposable, fast, chemical-free, portable, and self-observable for hemoglobin-related blood disorders like anemia by taking the input blood samples of less than 150 nL.

**Figure 15:**
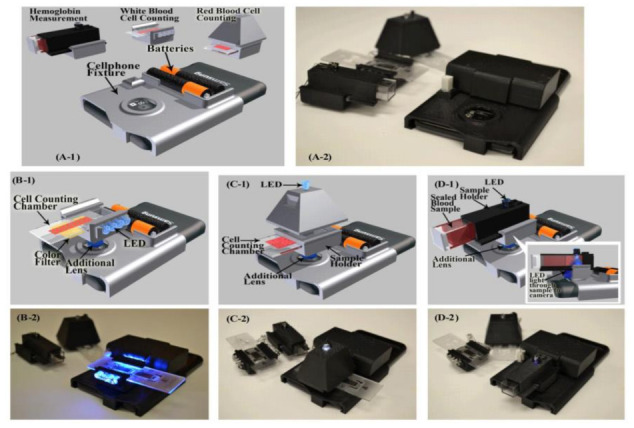
(**A1**,**A2**) Illustration and picture of our cell-phone based blood analysis platform. (**B1**,**B2**) Setup for white blood cell counting device. (**C1**,**C2**) Setup for red blood cell counting device. (**D1**,**D2**) Setup for a hemoglobin measurement device. Figure adapted with permission from [22].

**Figure 16:**
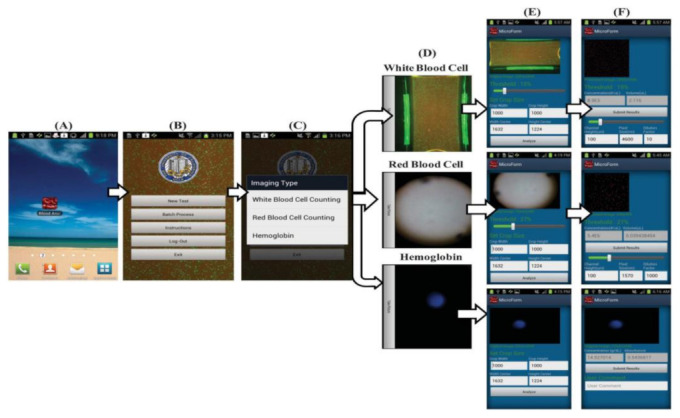
(**A**–**F**) Steps involved in the Android application to perform blood analysis and display results on a smartphone. Figure adapted with permission from [22].

Hongying Zhu et al. [22] developed a portable and low-cost cytometry imaging platform incorporated with the cell phone to measure hemoglobin density and white and red blood cells from human blood samples. These measurements help in clinical tests to analyze the health conditions and diagnose the different blood disorders like anemia and leukemia. A dedicated android application called “Blood Analysis” is designed to perform the operations. Two AA batteries are included in a base attachment and a universal port to hold three components for white blood cell counting, red blood cell counting, and hemoglobin measurements. Three separate test procedures are conducted individually to identify the density of hemoglobin and white and red blood cells, as shown in Figure 15. Its software approach is shown in Figure 16.

#### Summary

From the surveyed data, the disease diagnosis systems are classified into three categories used for hemoglobin measurement, glucose measurement and analysis, and whole blood analysis. Anemia, diabetes, and immunodeficiency are the primary diseases caused due to lack of hemoglobin, high glucose level, and red blood cell deficiency, respectively. For hemoglobin measurement, we studied three diagnosis systems, namely, hemoglobin measurement [11], blood analysis [22], portable chemical-free hemoglobin assay [21] with their functional parametric data available in their articles such as size, error rate and coefficient of variation, and the blood sample size in which the portable chemical-free hemoglobin assay [21] is efficient system due to its benefits like, requirement of least blood sample size of less than 1 µL, giving result in less than 8 s with an accuracy of 99%, and having advantages of such low cost, chemical-free procedure, and no requirement of medical personnel. For glucose measurement, we studied the systems, namely, glucose sensing [16] and whole blood glucose analysis [18], in which Glucose sensing [16] is a continuous measuring process by implanting a small steel-covered chip into the human body. In contrast, whole blood glucose analysis [18] is entirely an external application using a smartphone by taking a blood sample. Among them, complete blood glucose analysis [18] is efficient due to its smart functions, no internal nonspecific interactions, and also avoidance of implanting procedures that cause infection risk. For whole blood analysis, we studied the systems, namely, microfluidic cytometer [13], finger powered agglutination lab chip [14], blood analysis [22] in terms of blood sample size and functional capabilities in which blood analysis [22] is an efficient system as its blood sample size is of 10 µL, it provides the result in 10 s with an accuracy of 93%, and it is totally operated with a smartphone, which can transmit data wirelessly and thus leads to ease in remote sensing.

### 3.2. Disease Diagnosis Systems Related to Brain

Optogenetics technology is a demanding technology to implement light sensitivity on the different cell membranes using a genetic framework. This technology generated different techniques to investigate neural systems like the peripheral nervous system and brain. T. Tokuda et al. [23] developed an integrated microLED array with optical imaging functionality.

**Figure 17:**
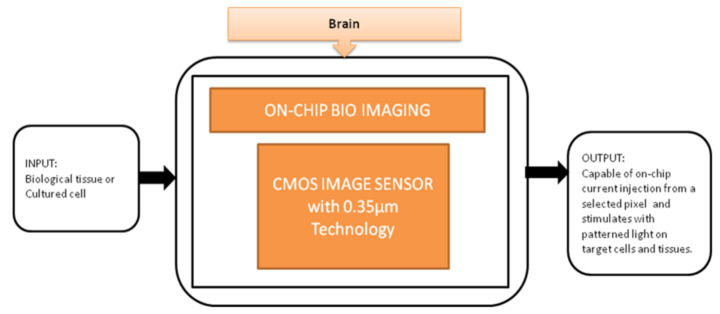
On-chip bio imaging.

The black-box approach of CIS-based neural interface device is illustrated in Figure 17. The brain slice is placed on the top of the surface on an integrated neural device, and light is stimulated from the multifunctional CIS, which is attached under the LED array. Single and multi-site stimulations are applied and obtained the on-chip image of the hippocampus slice of a mouse used to observe neural activities. This device is well suitable for on-chip brain imaging for in vivo and in vitro experiments.

**Figure 18:**
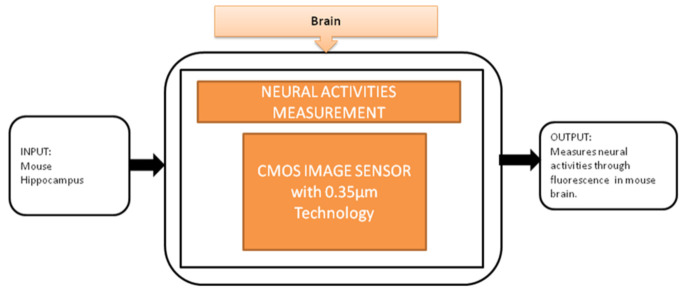
Implantable CMOS image sensor for neural activities measurement.

To observe the neural activities in the brain, fluorescence measurement is preferred over the electrical detection method because the fluorescence detection method is able to measure a large amount of data over a large area. Jun Ohta et al. [24] developed an implantable CMOS imaging device to record the neural activities in a mouse’s brain with minimal invasiveness. Its black-box approach is shown in Figure 18.

**Figure 19:**
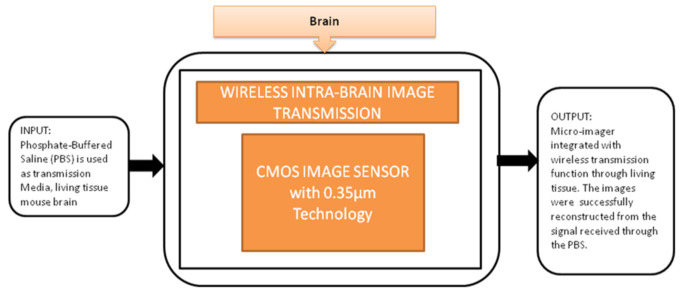
Wireless intrabrain image transmission using CMOS image sensor implanted in rat head.

This device can be directly implanted into the brain, and it has a completely dedicated CIS to excite the fluorescence on the flexible substrate. The head of the device is implanted into the mouse brain, and the remaining part outside of the brain is connected to the mini-PCB kept on the back of the mouse. A cable was connected from PCB to the mouse to move. In order to implant many chips in small animals, it is quite important to deliver the power and signals without wires because more wires can limit the free emotional behavior of the mouse. To meet this requirement, Kiyotaka Sasagawa et al. [25] developed an approach to communicate and transmit the power and signals for a short-range. He used the strategy of conducting properties of living tissues in animals for power communication, and its black-box approach is shown in Figure 19.

**Figure 20:**
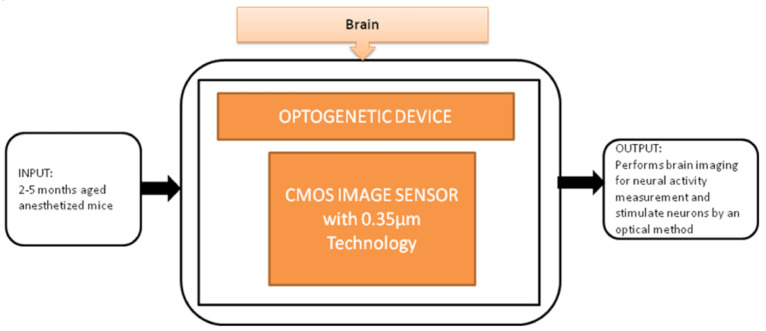
Implantable optogenetic device.

To control brain activities optically, optical stimulation techniques have been used, called Optogenetics. Makito Haruta et al. [26] proposed an implantable Optogenetic device for monitoring brain activities in awake animals with its black-box approach shown in Figure 20. This device incorporated with CIS can simultaneously perform brain imaging and optical stimulations and successfully evoke neural activities.

**Figure 21:**
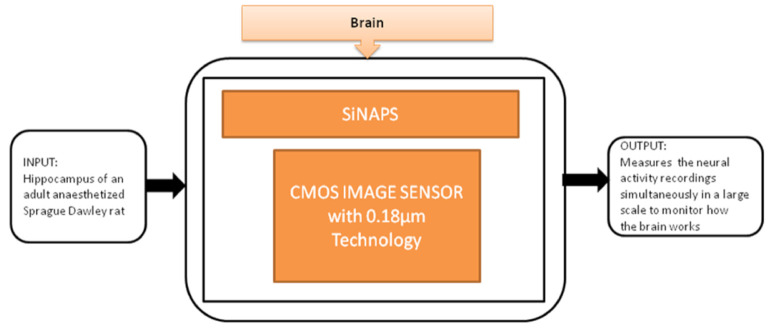
Simultaneous large scale neural recording SiNAPS probe for in vivo neuroscience research.

Gian Nicola Angotzi et al. [27] developed an implantable CIS probe for simultaneous large-scale neural recordings named “SiNAPS.” It was proposed to meet the requirements in neuroscience to analyze complex brain functions. Incorporating this device into procedures will allow capturing huge distributed cellular processing that characterizes and executes the brain’s complex functions, which will be useful in neural activity observations. Its black-box approach is shown in Figure 21.

**Figure 22:**
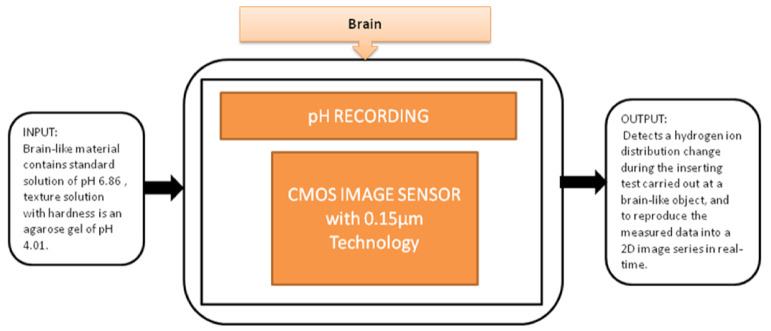
pH recording using CMOS image sensor.

You Na Lee et al. [28] proposed a pH image sensor for chronic recordings and obtaining the neurochemical signals that contain the spatiotemporal information. The image sensor can detect the hydrogen ions’ distribution change while carrying the insertion test at the brain, and then it reproduces the two-dimensional data from the measured data. The pixel circuit is a two-transistor (2-T) in the pixel circuit, which is advantageous. It has a low pixel pitch and high-density pixel array to enhance the visualization of spatial changes in the distribution of pH. Its black-box approach is shown in Figure 22.

**Figure 23:**
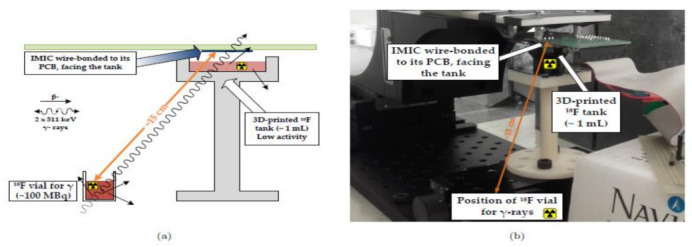
(**a**) Schematic of the experimental setup. (**b**) Physical setup. Figure adapted with permission from [29].

Heymes et al. [29] developed an IMIC circuit, a MAPS prototype (Monolithic Active Pixel Sensor), to incorporate the intracerebral positron probes, which are used for imaging the deep brain of the active rats as shown in Figure 23. This novel equipment of built-in probes using the CIS had tolerable overheating in the brain tissue of the rat or any other small animal. The author and their team are also working to generate the next generation of probes having reduced power dissipation with fewer pixels to be implemented in a real-time test.

#### Summary

It is mandatory to monitor the brain’s neural activities to detect the early stages of brain-related disorders like epilepsy, Parkinson’s disease, Alzheimer’s, and tumors. We studied the systems in terms of invasiveness, pixel size, and device size for neural activity measurement. Among them, neural activities measurement [24] is efficient due to its minimal invasiveness with a pixel size of 7.5 µm to image the neural activities. In addition, two types of image sensors, namely planar and needle type, are introduced. Intra brain image transmission [25] is efficient with its functional capabilities such as wireless transmission and deep brain imaging with the same pixel size of 7.5 µm, whereas, for simultaneous measurements, on-chip bioimaging [23], optogenetic device [26], and SiNAPS [27] are used, in which optogenetic device [26] is preferred due to its low pixel size and simultaneously performance of brain imaging and optical stimulation. Positron imaging [29] provides 100% detection efficiency with wireless transmission, but it affects physical activity due to its implanting facility.

### 3.3. Disease Diagnosis Systems Related to Skin

**Figure 24:**
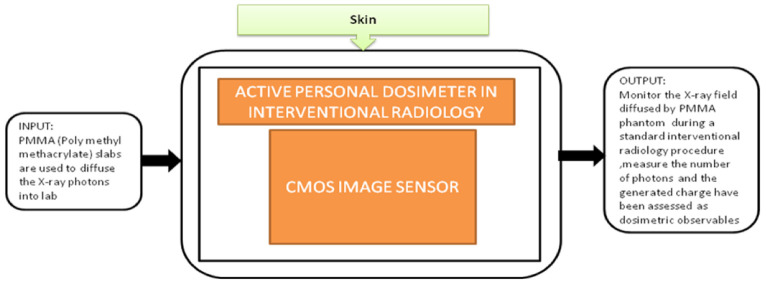
Active personal dosimeter in interventional radiology.

From the last decade, the research in biomedical healthcare instruments has been rapidly increased across the world. The investments in developing compact, low-power applications that could hold an extensive health information range are in great demand. During their professional works, staff members and interventional radiologists are frequently exposed to ionizing radiation in a low dose. These exposures can cause effects like aged skin, hand depilation, and radiodermatitis or may cause skin cancer. Elia Conti et al. [30] focused on one of these possible applications in Interventional Radiology (IR), a device to perform online monitoring of all the people involving in interventional activities. This portable device can measure the accumulated dose in real time and have an alarm to reduce the possibility of dose and collect the offline dose measurement results to create a temporal profile for the absorbed dose measurements. It gets correlated to staff-specific activities during the research. The black-box approach of the dosimeter is shown in Figure 24. This proposed system is a part of the Italian Real-time Active Pixel Dosimetry (RAPID) project framework.

#### Summary

This system is portable and helps in real-time monitoring of the staff involved in IR, thus helping the interventionists to estimate the radiation tolerance and plan further interventional procedures. However, the dosimeter is inadequate in terms of technology to meet all the types of interventional laboratory procedures.

### 3.4. Disease Diagnosis Systems Related to Intestines

**Figure 25:**
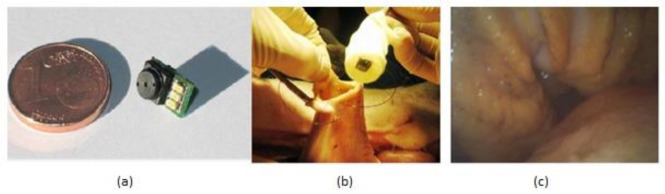
(**a**) Camera module prototype. (**b**) In vivo experiments in pig’s stomach. (**c**) Acquired image of recorded video. Figure adapted with permission from [31].

The gastrointestinal tract screening started in the past few years and still is widely used now with the help of an endoscope, which is sent into the human body through the natural orifice. It is a painful process and needs anesthesia to be given to the patient while doing it. However, the latest developments in wireless capsular endoscopy make this a noninvasive process, and it has become a completely painless procedure. Covi et al. [31] proposed a camera module system to be used in endoscopic applications like endoscopic capsules. The prototype of the camera module is shown in Figure 25.

Experiments were made on a porcine model to evaluate the camera module performance in terms of its illumination and image quality in real time. This camera module is connected to the capsular shell, which is wired with a computer and sent into the pig stomach to visualize the required regions of interest, and the video was captured about gastroscopy for 30 min, which can be seen in Figure 25b,c. The quality of video of captured images is good, sufficient for the physicians to make a suitable diagnosis. Due to its low manufacturing, this module can be used for disposal applications.

The gastrointestinal tract’s noninvasive screening and diagnostic assessment can be made using wireless capsule endoscopy (WCE). The first capsule endoscopy entered the market in 2001. This capsule has size and shape of a pill, and it consists of a small, low-resolution camera with LED illumination, a radio transmitter, and two batteries. This capsule passes through the intestines and sends the images to the data recorder attached to the patient.

**Figure 26:**
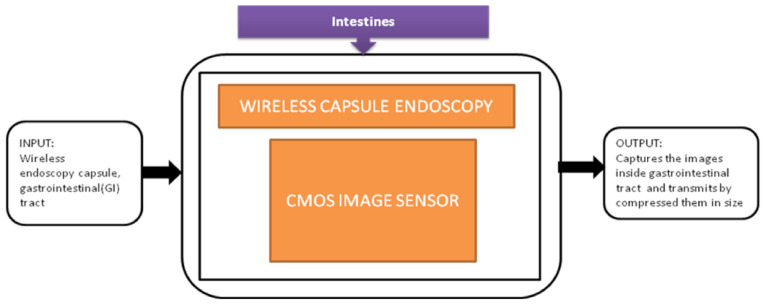
Wireless capsule endoscopy.

The major activity of research in wireless capsule endoscopy focuses on the automatic identification of regions in the gastrointestinal tract with unusual conditions like bleedings and tumors. The incoming generation of this wireless capsule endoscopy features real-time manipulation remotely to verify any damage in an organism’s tissue. Narrow-band imaging enhances the imaging of the mucosal microvascular structure. To transmit the data images under real-time manipulation of wireless capsule endoscopy, it requires efficient image compression. Pawel Turcza et al. [32] developed an image processing system for wireless capsule endoscopy in which a dedicated image coder is inserted. The black-box approach of wire capsule endoscopy is shown in Figure 26.

**Figure 27:**
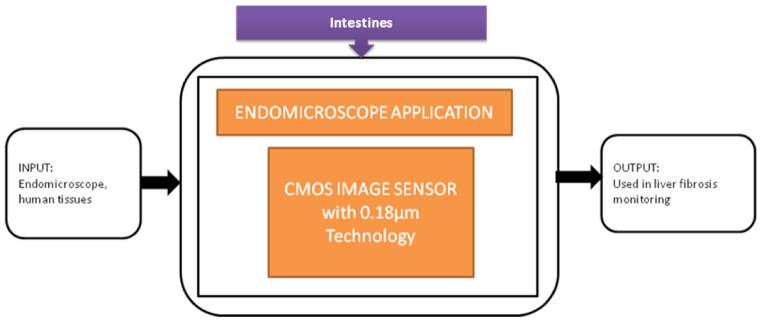
Endomicroscopic application with self-energy harvest technology.

Tong xi Wang et al. [33] designed an ultra-low power CIS particularly used for endomicroscopy applications. This image sensor has a dual operation mode, which leads to self-powering internally. The pixel can also be converted into a solar energy cell to harvest the energy for the sensor’s operation, and its black-box approach is shown in Figure 27.

#### Summary

Among the three systems discussed/reviewed earlier disposable endoscopic application [31] is having the advantages of providing highly efficient illumination, acceptable image quality with a FOV (field of view) value of 60 degrees, and disposable. However, it is still a painful procedure that requires the patient to be anesthetized. Wireless capsule endoscopy [32] has image compression capability with low power consumption and wireless transmission, but this device does not work for long-duration surgeries and need an external power supply. Endomicroscopic application [33] is also a low-power operating device with on-chip image data analysis, but the sensitivity is poor in low light conditions.

### 3.5. Disease Diagnosis Systems Related to Eyes

**Figure 28:**
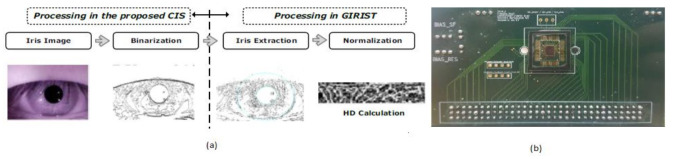
(**a**) Iris recognition process with proposed CIS. (**b**) CIS chip on printed circuit board (PCB) [34].

From the last 10 years, different information technologies were developed, and the importance of security systems is also increased for mobile phones. The level of security in biometrics, involving voice, retina pattern, fingerprints, etc., is quite substantial since the chance of making duplicates is significantly less. The development of Iris detection has been rapidly increasing to unlock mobile phones like iPhone X. The Iris detection algorithm’s traditional process involves image acquisition, Iris image enhancement, binarization, and recognition. However, the output of this process is CIS that yields multi-bit data. So, Soo Youn Kim et al. [34] proposed a single-bit CIS, which can show the Iris recognition process shown in Figure 28.

**Figure 29:**
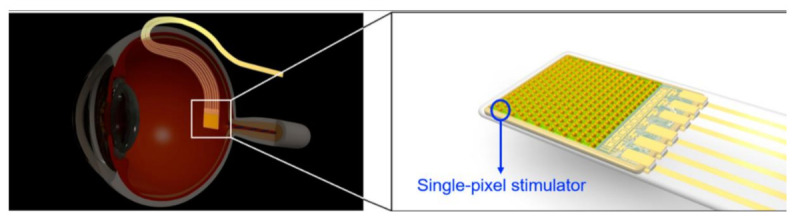
Subretinal prosthesis [35].

Retinal implants make great efforts to restore patients suffering from retinal diseases such as age-related macular degeneration and retina pigmentosa. Concerning anatomy, a retinal prosthesis is classified as subretinal, epiretinal, suprachoroidal devices. Among the classifications, the subretinal implant is providing a high pixel density of 1600 pixels. Hosung Kang et al. [35] developed a stimulator with low mismatch ability integrated with photodiode used for a subretinal prosthesis, as shown in Figure 29.

**Figure 30:**
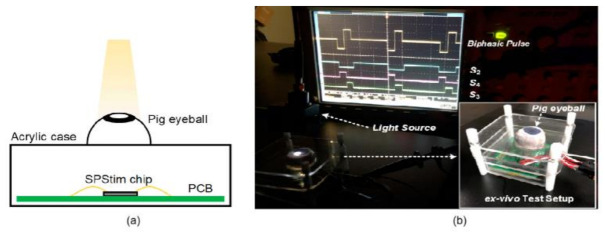
(**a**) Pig eyeball. (**b**) Ex vivo experiment setup using a dissected pig eyeball [35].

Subretinal Prosthesis Simulator called as “SPStim” demonstration is done using pig eyeball, which was shown in Figure 30a, and its ex vivo setup can be seen in Figure 30b. This “SPStim” is well suitable for subretinal implants, and it has very little power consumption.

**Figure 31:**
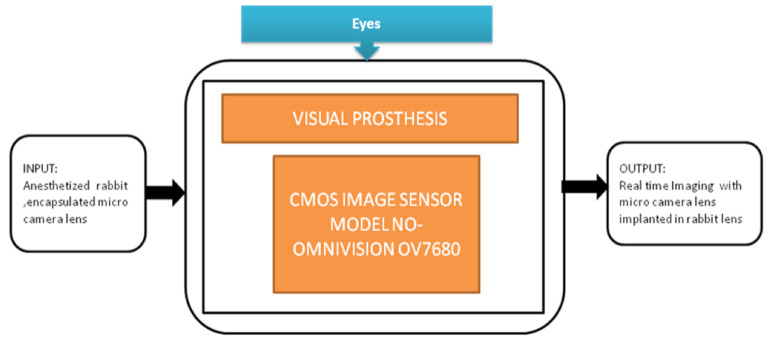
The implantable micro camera system in the visual prosthesis.

Image sensors are acting as additional optical units that can capture scenes in the latest retinal implantations. Hundreds of thousands of patients have started steadily losing their vision or getting blind because of retinal degenerative diseases. Chaunqing Zhou et al. [36] proposed an implantable imaging system for a visual prosthesis. A micro camera is developed for this purpose and devised to fit in the rabbit’s lens capsule, and biocompatible silicon is used to encapsulate it. For the clinical procedure, 12 micro cameras having CIS into 12 eyes of 12 rabbits were implanted. Its black-box approach is shown in Figure 31.

### 3.6. Disease Diagnosis Systems Related to Heart

Due to drastic development in integrated circuit manufacturing, wearable devices like smart jackets and smart bands are used widely to assess human heart and motion detection with the help of electrodes. Inertial motion sensors are integrated into them. However, these smart devices are causing potential risk due to leakage in current and discomfort using them for a long time. Due to this reason, people with sensitive skin or neonates are suffering from rashes and allergies after wearing smart wearable devices for a longer time.

**Figure 32:**
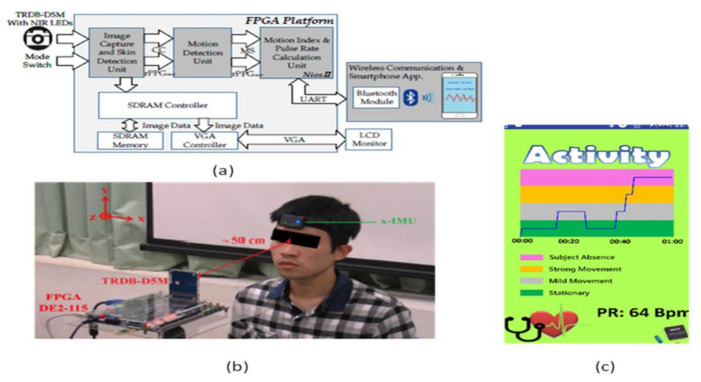
(**a**) System architecture. (**b**) Experimental setup. (**c**) Data display on Android application in the smartphone [37].

To overcome this problem, contactless sensors are being introduced. Yu-Chen Lin [37] et al. developed a contactless pulse rate detection and motion status monitoring system. The motion status is obtained by complexion tracking with a motion index developed to eliminate motion artifacts to enhance pulse rate measurement accuracy. Near-infrared LEDs are attached to measure in dark conditions. A switch is used to enable the dark mode and brightness mode detections. An experimental trial is conducted on 10 volunteer people, including three females and seven males between 22 and 30 years with no earlier heart-related disorders. The people sit in front of the system with a distance of 50 cm for pulse rate detection. The smartphone is connected to the developed system using Bluetooth, and results are displayed on the Android application in the smartphone shown in Figure 32.

### 3.7. Disease Diagnosis Systems Related to Lungs

**Figure 33:**
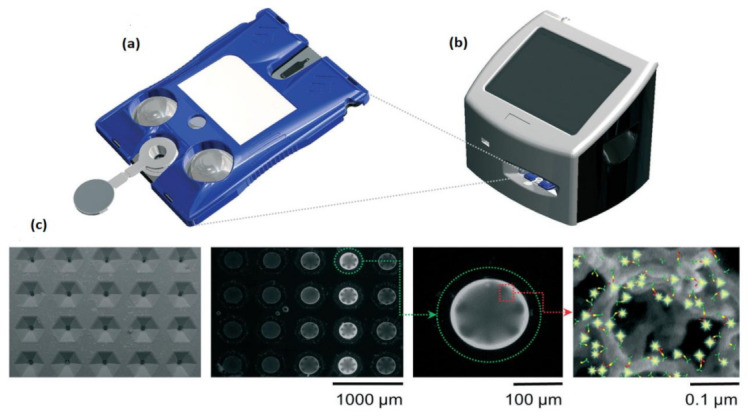
(**a**) The cartridge used in programmable bio nanochip (p-BNC) assay system. (**b**) Portable instrument. (**c**) SEM image, a fluorescent micrograph of bead sensors. Figure adapted with permission from [38].

Around 97 million people are affected, and over 2 million people are died due to COVID-19 (coronavirus disease) worldwide. More significant outbreaks occurred in the United States, Italy, Spain, India, Brazil, Russia, etc. The symptoms of this disease are predicted accurately. The infected people may have a cough, fever, shortness of breath, muscle aches, fatigue, and causes pneumonia attack that severely leads to acute respiratory distress syndrome and causes death. Due to unfortunate outbreaks, hospitalizations are increased in huge numbers, and the overwhelming need for intensive care units is necessary to save the patients from dying. Michael P. McRae et al. [38] developed an integrated care COVID-19 clinical decision supporting system to find the severity score of the COVID-19 patients to prioritize their care and resources for treating them in which a disposable programmable bio nanochip (p-BNC) is used to determine the severity of COVID-19 as shown in Figure 33.

**Figure 34:**
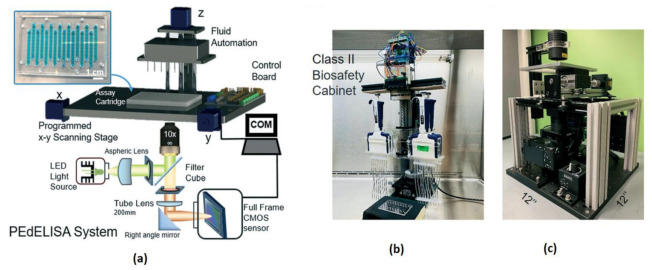
(**a**) Pre-equilibrium digital enzyme-linked immunosorbent assay (PEdELISA) system-photo image. (**b**,**c**) PEdELISA system schematic in a bio-safety cabinet. Figure adapted with permission from [39].

Most of the complications in COVID-19 are caused by a cytokine storm. It triggers the immune system to include the inflammatory proteins known as cytokines, which kill tissues and organs in a human body. Yujing Song et al. [39] developed a clinical application called “PEdELISA (pre-equilibrium digital enzyme-linked immunosorbent assay) microarray” to monitors the cytokines in the severely affected COVID-19 patients who are admitted to intensive care units in a hospital. The developed system is having a fast four-plex measurement of cytokines in the serum of COVID-19-affected people, and the schematic diagram is shown in Figure 34.

Lack of risk assessment of COVID-19 airborne particle transmission in public environments like classrooms, elevators, restaurants, and supermarkets leads to uncertainties in their preventive measures to control the spread of COVID-19. Siyao Shao et al. [40] conducted a risk assessment of exhaled particles from different people in various environmental settings, which are becoming local hotspots for tremendous outbreaks of COVID-19. Different breathing patterns are being recorded from 8 healthy participants between the ages between 21 and 29 with a breathing rate of 76 beats per minute, nose inhales for two beats, while mouth exhale for three beats. The breathing patterns are followed the Schlieren imaging breathing technique for all the experiments.

**Figure 35:**
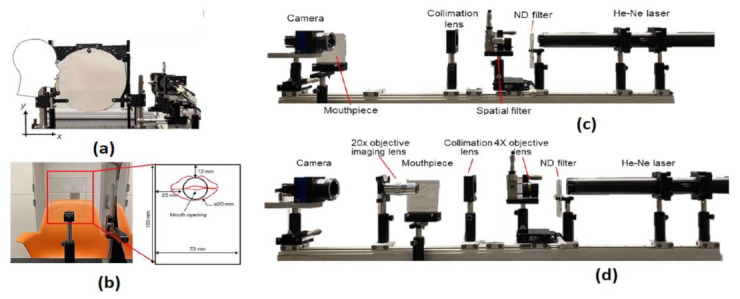
(**a**) The head position of the participant. (**b**) Breathing direction of the participant. (**c**) 1× DIH measurement system. (**d**) 20× DIH measurement system. Figure adapted with permission from [40].

**Figure 36:**
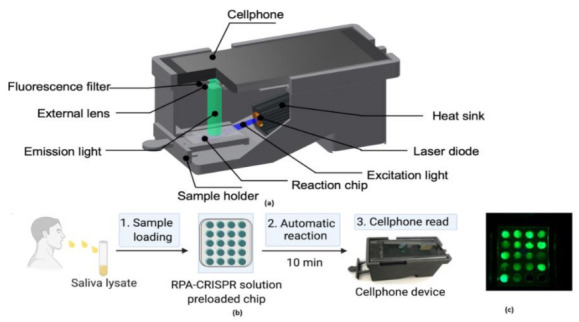
(**a**) The 3D-printed schematic of smartphone fluorescence reader (**b**) Saliva-based CRISPR Fluorescence Detection System (FDS) Assay procedure. (**c**) Capture images of CRISPR-FDS Assay [41].

The digital inline holography (DIH) technique is used to assess the individual microparticles in a sample volume. Moreover, 1X magnification digital inline holographic imaging system is involved in capturing the exhaled particles in size from 10 to 50-µm range; 20× magnification digital inline holographic imaging system in capturing the exhaled particles, which are in size from 3 mm to 2-cm range, is shown in Figure 35. To improve the COVID-19 disease control efforts, Bo Ning et al. [41] developed a compact saliva-based COVID-19 assay using a smartphone without using the laboratory equipment that provides the results in 15 min. This procedure is easier than RT-PCR test, which is currently used to perform the COVID-19 test. The assay used in the proposed test utilizes Clustered Regularly Interspaced Short Palindromic Repeats (CRISPR)Cas12a activity triggers the virus DNA, and this CRISPR reads COVID-19 from the patient’s saliva and nasal swabs since the RNA level in both places is similar. The smartphone fluorescence microscope device is used to read the CRISPR fluorescence detection system assay to analyze saliva from COVID-19-affected patients, shown in Figure 36.

#### Summary

Among the four systems reviewed, COVID-19 severity detection [38] uses the statistical learning algorithm and provides the severity score result in 16 min to give the suitable treatment and predict mortality with accuracy. Still, some of the input biomarkers yield redundant data that cause different results. COVID-19 cytokine storm monitoring [39] maintains high speed and sensitivity compared to existing in analog label-free point-of-care systems, but the system’s capacity is limited to 16 samples. COVID-19 risk assessment [41] provides the possibility of spreading of COVID-19 droplets with good accuracy. It proved that multiple ventilation could reduce the spreading. COVID-19 saliva test [40] is having a good correlation, portable. It provides the results in 15 min with 98.7% accuracy and is user friendly as no medical experience is needed, but the error occurs due to lysing of saliva for analysis.

### 3.8. Disease Diagnosis Systems Related to Bones

**Figure 37:**
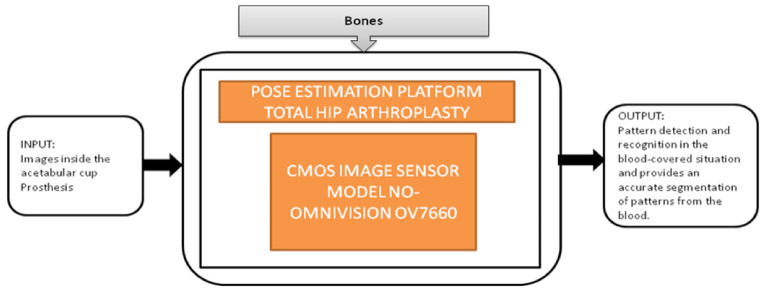
Pose estimation platform for total hip arthroplasty.

Nowadays, people are getting old, and at their above-average age, people are getting through arthroplasty surgery for their damaged hip joints to be replaced. Various visual aided systems were introduced in order to reduce the risk factor earlier. Syed Mudassir Hussain et al. [42] proposed an image sensor that can replace a bulky camera from the pelvis’s femoral head, which saves most of the system’s power. A new technique was introduced to identify every pattern from the situation covered with blood, which generates the respective binary ID. The vision-based smart trail with test setup is also represented, and its black-box is shown in Figure 37.

**Figure 38:**
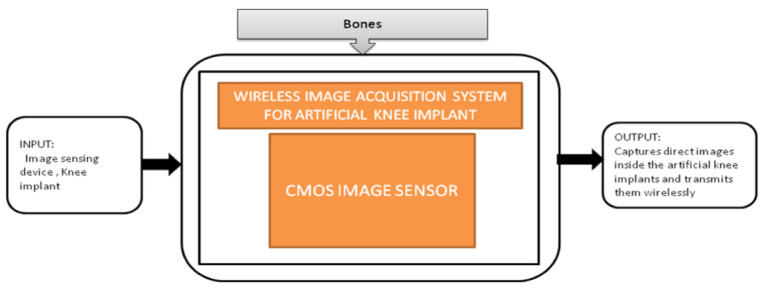
Wireless image acquisition system for knee replacement implant in total knee arthroplasty.

Nowadays, the population which is suffering from arthritis is growing very rapidly. By 2030, in the United States, the affected people above the age 65 and older would cross 41 million. Among those people, knee arthritis affects the quality of life in a depreciation manner. The surgical treatment for this device is total knee arthroplasty (TKA), in which an artificial implant will replace the affected knee joint. Shaolin Xiang et al. [43] proposed a wireless acquisition system that can be used in TKA surgeries. This system will directly provide the inside knee implant images to the workstation wirelessly using a wireless data logger. Its black-box is shown in Figure 38.

#### Summary

The two systems, pose estimation platform for total hip arthroplasty [42] and knee implants [43], are playing a major role in assisting doctors by providing real-time images during surgeries for better accuracy and quality. In addition, using these devices reduce the chances of failures and side effects in surgeries. These two devices can work in a low-light environment also.

### 3.9. Disease Diagnosis Systems Related to Bacteria Cells

Biofilms are nothing but a pack of bacterial cells with extracellular polymer substances automatically generated by bacteria by exposing them to rough atmospheric conditions. These biofilms are majorly present on surfaces of pipes, dead tissues, and medical instruments used for in vivo experiments. The effects of biofilms include medical device damage and degradation of the drinking water quality. It is difficult to remove these biofilms from water, medical instruments, and pipes.

**Figure 39:**
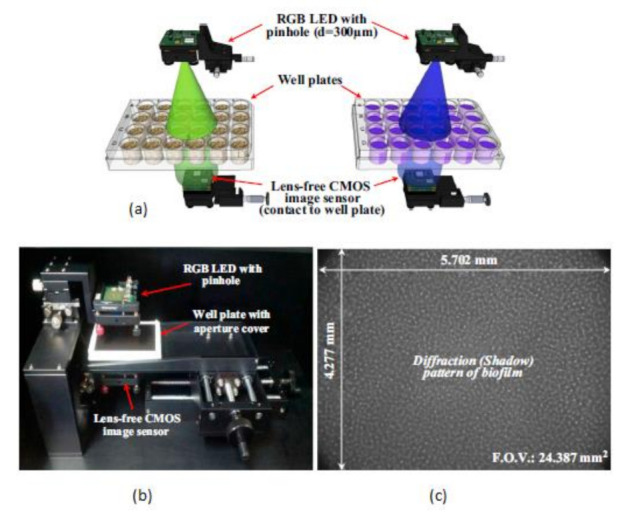
(**a**) RGB (Red Green Blue) LED illuminations. (**b**) Lens free CMOS image sensor-based biofilm detection. (**c**) The pattern of biofilm. Figure adapted with permission from [44], Elsevier, 2014.

**Figure 40:**
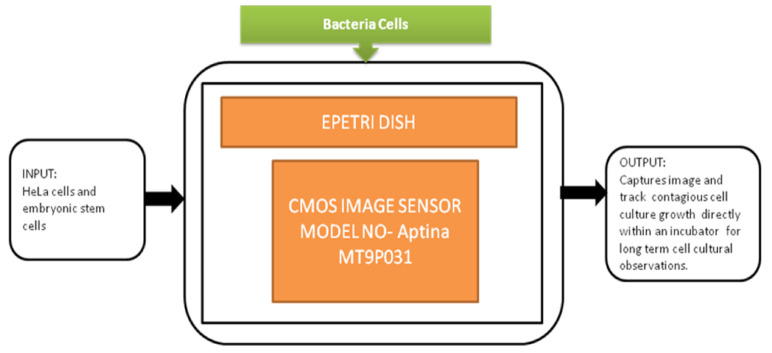
ePetri dish.

This biofilm’s major effect is that it causes infection by *Pseudomonas aeruginosa* and makes pneumonia untreatable to the patients with less immunity, which can lead to death. Yeon Hwa Kwak et al. [44] proposed a lens-free CIS, which is low cost and easily identifies colorimetric changes in biofilms concentrations. This lens-free CIS-based biofilm detection platform is a simple and less costly method with a wide range of possibilities to be used in medical and environmental biofilm detections, shown in Figure 39. Guoan Zheng et al. [45] developed an on-chip cell imaging platform based on SPSM (subpixel perspective sweeping microscopy). The prototype of ePetri is kept in a square plastic box with a glue and polydimethylsiloxane (PDMS) layer that is used to cover the prototype, and its black-box approach is shown in Figure 40. This layer is thin and prevents cultural evaporation during the exchange of carbon dioxide between the plastic box and cover.

**Figure 41:**
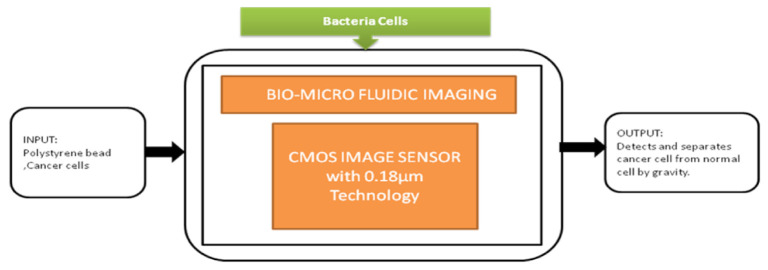
Cancer cell separation by gravity from the normal cell through biomicrofluidic imaging device.

The smartphone screen is used as a light scanner. It is a lensless microscopy imaging system that can capture confluent cell structures through random light resources. It can also give high-resolution imaging and can be used as an emerging tool for in vitro cell observations. Nowadays, flat-panel images are the common detectors in biomedical applications in which large imaging areas with high frame rates are required. Fluoroscopy, mammography, proteomics, and image-guided radiotherapy need large imaging areas having real-time frame rates. However, flat-panel imagers are having drawbacks such as large pixels, low frame rates, and high noise. To overcome these drawbacks, active pixel sensors came into the picture and received good fame by having features like low noise, high frame rate, and high pixel resolution, low power consumption, and low manufacturing cost. Esposito et al. [46] developed a new radiation-hard monolithic active pixel sensor called (Dynamic range Adjustable for Medical Imaging Technology) DynAMITe. This imager consists of two separate resolutions with different saturation performances and different noise in the same pixel array. This design had tremendous advantages in various biomedical applications, which need a high dynamic range, high frame rate, and large pixel area. Lensless imaging systems play a crucial role in biomedical imaging applications like cancer cell detection and medical tomography, Optofluidic microscopy, etc. Previously, microfluidic CIS has a limitation of low sensitivity. However, pixel size should be considered to improve the sensitivity, but it reduces the resolution. Wang et al. [47] introduced a super-resolution CIS for biomicrofluidic imaging applications. The authors conducted an on-chip single frame super-resolution image processing algorithm also. Polystyrene bead is a colloid particle used to simulate the cell’s behavior in a biomicrofluidic system, and its black-box approach is shown in Figure 41.

**Figure 42:**
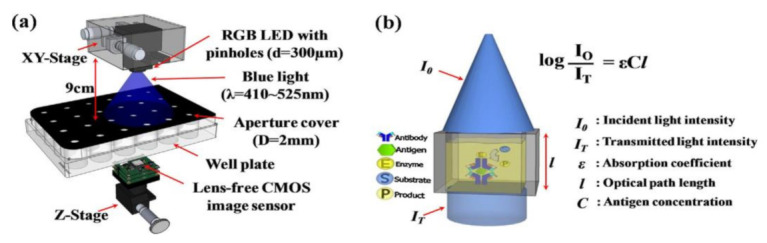
(**a**) CIS-based ELIZA detector. (**b**) Absorbance measurement and ELIZA assay. Figure adapted with permission from [48], Elsevier, 2014.

Junhe lee et al. [48] proposed a portable and affordable lens-free CIS-based bacterial cell detection platform, which can measure colorimetric light signals enzyme-linked immunosorbent assay process ELISA Process. This process had become the famous process to detect the molecules and thereby analyze the change in the colorimetric degree of a molecule in immune reactions in almost all biomedical research. This ELISA detection platform uses multimillion pixel arrays, which are CIS instead of single photodiodes shown in Figure 42.

**Figure 43:**
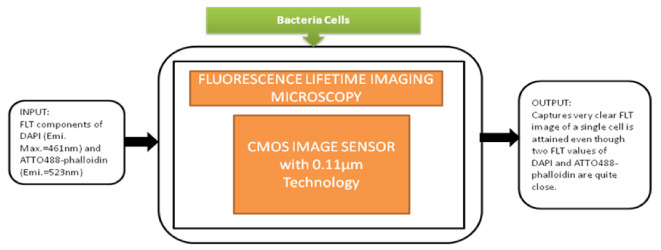
Fluorescence lifetime imaging microscopy is used to capture color fluorescence lifetime images of HeLa cells with DAPI nucleus.

This detection system had a great role in prominent applications like blood analysis, environmental monitoring, and pathogen detection. In medicine and life sciences, the fundamental methods are fluorescence-based time-resolved analysis techniques. In those methods, fluorescence lifetime imaging microscopy (FLIM) is a promising measurement process in biomedical applications. Min Woong Seo et al. [49] proposed the sub-nanosecond time-gated four tap lock-in pixel CMOS image sensor with an in-pixel pulse generator (PG) to be used in fluorescence lifetime imaging system. The i black-box approach is shown in Figure 43.

**Figure 44:**
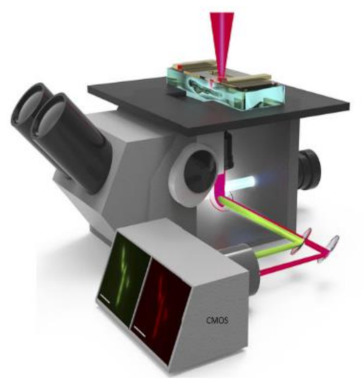
Temperature jump fluorescence Imaging microscope [50].

Ruopei Feng et al. [50] proposed a pipeline that joins the meganuclease-mediated transformation with fluorescence detected temperature jump microscopy to capture fast dynamics of biomolecules in living multicellular organisms with single-cell resolution. The demonstration is also done on folding kinetics and stability of the fluorescence resonance energy transfer-labeled glycolytic enzyme phosphoglycerate kinase in individual cells of four zebrafish tissues. CMOS camera (Figure 44) is used to record the millisecond time resolution moves of kinetics in the living zebrafish cell, i.e., keratinocytes, eyelids, notochord, and myocytes.

Another push of smartphone technology into personal healthcare systems is using tissue and cell imaging applications to identify the accurate disease and health information.

**Figure 45:**
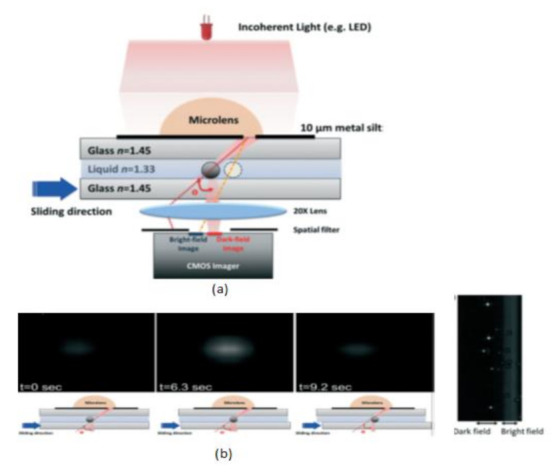
(**a**) Scattering image-based cytometer (**b**) Images of traveling bead over the sensing area in different time intervals from dark field to bright field. Figure adapted with permission from [51], Royal Society of Chemistry, 2014.

**Figure 46:**
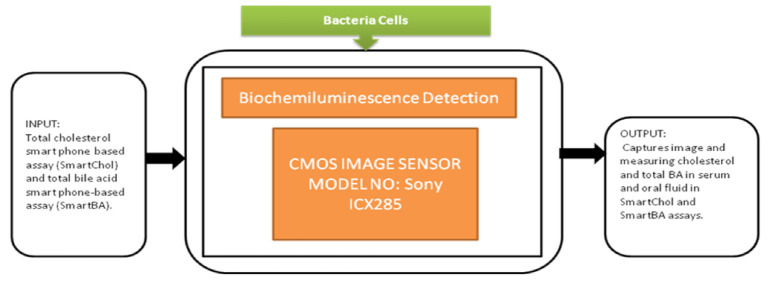
Biochemilunimescence detection.

Tsung-Feng Wu et al. [51] proposed a unique approach to develop a good-quality cytometer compatible with smartphones, shown in Figure 45. For the demonstration, HEK293 cells and isolated white blood cell samples, inserted between the two glass cover strips, are taken to analyze the cytometer. The CIS captured the images with a frame rate of 10 fps. It is a label-free cell analyzer that is able to detect the internal structure of cells, and it is well suitable for point-of-care diagnostics. Aldo Roda et al. [52] presented a smartphone-based biochemiluminescence detection, a portable chemistry platform for point-of-care analysis. This system can capture and measure the biochemiluminescence attached with biospecific enzymatic reactions in biological fluids like oral fluid and blood. Its black-box approach is shown in Figure 46.

The 3D printer is used to design and fabricate the prototype accessory to be attached to the smartphone. This prototype had dual functions to act as a dark box to cover from ambient light and carries the mini cartridge used for diagnostic purposes and chemical fusions. This is an excellent instrument that could be used in fast liver function evaluations in dogs and cats.

#### Summary

From the reviewed data, Biofilm detection [44] is cost-effective and using lens-free CMOS imaging technology with a large field of view 25 times larger than the general optical microscope. ePetri dish [45] captures the cell growth of contagious cell cultures. It is available at an affordable price, which is disposable and recyclable, but the system is very slow and limited to non-fluorescence imaging. DynAMITe [46] has two-side buttable technology with two different pixel sizes, which yields more pixel size imager in biomedical imaging. It offers high frame rates and high spatial resolution. Biomicrofluidic imaging [47] can separate the cancer cells from normal cells and adapt to pixel size from high to low with high frame rates. Still, it needs to improve the low light sensitivity, which is affecting the resolution. ELISA detector [48] is noise-free and low cost with compact in size. The pixel size is significantly less sufficient to image the bacteria cell detection, but a discontinuity in light transmission will occur at some concentrations due to biological procedures and dilution errors. FLIM [49] is having fast data acquisition with low noise and error, but it comprises higher voltages and complex circuits. Quantifying protein dynamics [50] is available to perform both in vivo and in vitro tissue type experiments. The proposed pipeline methodology helps in photodynamic cancer therapy, but this system cannot obtain individual cells’ time-resolved data within live vertebrate organisms. Intracellular imaging and biosensing [51] is the first device to provide significant information about cell features like size, shape, and life cycle. Still, the magnitude intensity difference is beyond the dynamic range. Biochemiluminescence detection [52] performs frequent and noninvasive bile acid monitoring with a coefficient of variation from 5% to 12% within a time period of 2 days.

## 4. Data Extraction and Evaluation

The extracted data from the conducted literature study meets our criteria for the review. The data consists of Table A1 (Appendix A) containing the information from our study (e.g., title, year of the article, CIS model/camera module; type of fields included e.g., medical, biomedical, and implantable), application name/target, and CIS technical specifications included (e.g., pixel size, resolution, pixel pitch, sensitivity, dynamic range, signal to noise ratio, fill factor, area, and power and frame rate). The data for all studies are extracted independently by two authors S.B.S. (Suparshya Babu Sukhavasi) and S.B.S. (Susrutha Babu Sukhavasi), under the supervision of author K.E. and discussion with other authors (A.E. and S.A.).

In this section, the basic evaluation of each device’s vital parameters is given as per our survey. We took five parameters as described in Table 1. We have done a quantitative evaluation for the articles reviewed based on the key parameters in terms of their medical improvement for the diagnosis of various diseases.

The device assisting the medical diagnosis requires a lot of parameters, among them, the parameters like the testing method that involves in vivo or in vitro or both the testing methods, works real time or not, remote sensing possibility for ease of diagnosis helps in disease diagnosis in various organs of the human body and pain scale for patients’ tolerance check; these parameters are playing a crucial role in data evaluation.

The parameters that we used for evaluation are to be considered as basic fundamental parameters for medical device selection in diagnosing the human body. Uniform weightage of 10.0 is assigned to all parameters initially and varies concerning their available options. For example, we gave the weightage of 10.0 for the glucose-sensing system because the system can perform testing in both in vivo and in vitro methods, where an 8.0 value will be assigned for the system doing in vivo testing method, and a 6.0 value will be assigned for the system doing in vitro testing method. The value 10.0 is assigned for a system having remote sensing ability, and value 5.0 is assigned for systems with no remote sensing. The value 10.0 is assigned for the system that can be implemented in real time and value 5.0 is assigned for the system with no real-time implementation. We used (numerical rating scale) NRS pain scale [53] for pain assessment and to rate the pain level of the patients in between 0 and 10 numerical values such that the value 0 refers to none, the value 1–3 refers to mild pain, the value 4–6 refers to moderate pain, and the value 7–10 refers to severe pain. Every single parameter has its own impact on the diagnosis procedure, and the score is given parameter-wise based on the available data in the literature survey.

Using the below normalization formula (Equation (1)), we have calculated the total score for every system based on Table 2. The total score of every system in Table 2 provides a brief evaluation of the medical devices’ abilities.

(1) $$\mathrm{Total}\;\mathrm{Score}=\overset{N}{\underset{k=0}{\sum }}\frac{10P_{k}}{N}+2,$$

where *k* is the particular parameter (e.g., pain level) used in the evaluation, *N* is the total number of parameters of each system, P*_k_* represents the parameter value of each system_,_ and the value 2.0 is added as a constant to the equation to help in highlighting the system’s evaluation from its parameter score.

**Figure 47:**
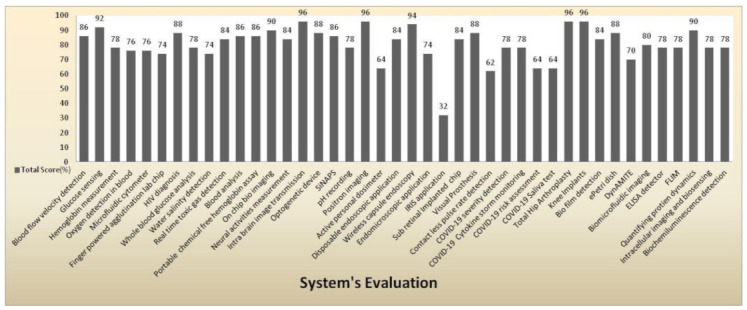
System evaluation represents the total score of every medical system.

Figure 47 shows a clear pictorial representation of each system with a total score to every system involved in our literature survey. The systems with a higher score of more than 90% indicate the medical system is advanced, can be accessed remotely, implemented in real time with good efficacy, and has a reasonable pain scale to perform disease diagnosis. IRIS application obtained a very less score of 32% with a zero pain scale due to neither involving in any diagnosis procedures nor performing any testing methods than other systems. The average score obtained by most of the systems is in the range between 74% and 78%. Intra brain image transmission, positron imaging, total hip arthroplasty, knee implant systems scored 96% as they had remote sensing capabilities and real-time implantation and involved testing methods to perform disease diagnosis.

## 5. Discussion

After reviewing the blood-related disease diagnosis systems, we found the most potent disease diagnosis systems related to blood is portable chemical-free hemoglobin assay [21] due to its least blood sample size of less than 1 µL, giving result in less than 8 s with an accuracy of 99% and having advantages of its low cost, chemical-free procedure, and no medical personnel is needed. Blood analysis [22] can provide the density of RBC (red blood cells) and WBC (white blood cells), along with hemoglobin measurement with an accuracy of 93% and provides the result in 10 s by taking the blood sample of 10 µL. The most potent disease diagnosis system related to the brain is intra brain image transmission [25], which covers the brain’s surface and imaging the cross-section with very low invasiveness compared to other systems and portable size of less than 1 mm in volume. The potent system related to intestines is endomicroscopic application [33] due to its energy-harvesting capability inside of an imaging pixel. It operates in two modes: self-powering modes and imaging pixel mode, which causes the system to consume ultra-low-power of 6 microwatts. As per our study, we consider the potent system related to lungs is COVID-19 severity detection [38] because, through this system, the disease severity of the patient can be detected and thereby predict the mortality, from that, the decision can be made whether the patient can be quarantined or needs to be treated in ICUs (intensive care unit). As per the survey, the most powerful system related to bacterial cells is biofilm detection [44] that uses lensFree imaging technology with an ultra-large field of view and zooming capacity of 25 times larger than 100× optical microscope with three times more stability.

From the surveyed parametric data of Appendix A, we observed that the pixel pitch plays a crucial role in blood-related disease diagnosis. The pixel pitch of 7.5 µm is enough to perform blood analysis. In contrast, the pixel pitch range of 2.5–4 µm is required to identify and separate red blood cells and white blood cells from the blood sample. For brain-related disease diagnosis, while designing the device, the LEDs used to stimulate causing the power dissipation are to be controlled in case of brain-related disease diagnosis. This causes heavy stains in blood, which interrupts the imaging of the brain for neural measurement activities. The array resolution should be high so that more details will be captured related to blood vessels and flow of blood in the brain. The device’s size should be miniaturized so that it will not add more weight to the brain for diagnosis. An imager’s area to meet the design criteria for implant measurement diagnosis is to be in needle type, and planar type design with a very slight thickness width range of 0.2–0.5 mm can easily penetrate the brain hippocampus and cause fewer stains inside for brain-related diagnosis. The pixel pitch of 2.2 µm is well suited for compact devices for cell screening, and imagers with high resolution can be used to compare with a 100× microscope, which is efficient to observe more minute details about the cell growth and lifecycle evaluations. In addition, we observed that some of the papers were provided with a few parameters of CMOS image sensors involved in the disease diagnosis systems. Still, the system capabilities are clearly explained, which helped us in evaluating the systems.

### CMOS Image Sensor Models

**Figure 48:**
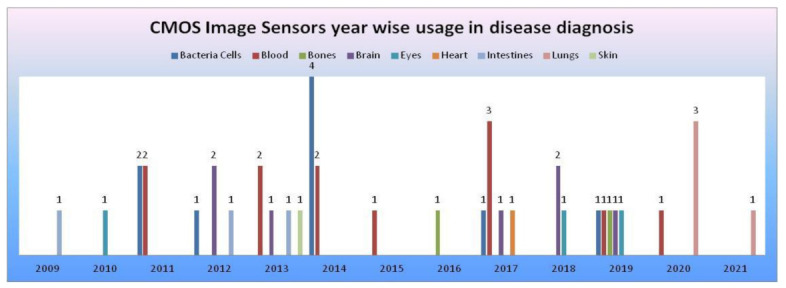
CMOS image sensors year-wise usage in disease diagnosis according to survey data, where the *x*-axis represents years and the *y*-axis represents the number of CMOS image sensors involved in disease diagnosis of human body organs.

From the data collected in our literature survey, we have mapped the CMOS image sensors models, camera modules embedded in medical devices performing disease diagnosis for the human body’s vital signs, and organs in Table 3. CIS manufactured with 0.18 and 0.35 µm technologies [54] were being used in most disease diagnosis systems. Samsung smartphone was used in most smartphone-based disease diagnosis systems. CMOS image sensors year-wise usage in disease diagnosis systems according to survey data is shown in Figure 48.

## 6. Conclusions

This paper presented a systematic review and parameter-based evaluation of CMOS image sensors incorporated disease diagnosis systems from the last 12 years. We reviewed the systems and identified the advantages and disadvantages of every system involved in the survey. Every system is evaluated by considering the parameters related to its capabilities, implementations, and testing methods involved. The most potent systems with prominent capabilities and functions are discussed, and organ wise involved CMOS image sensor models are mapped. Despite CMOS image sensors’ existence in imaging technology for the past two decades, this is sophisticatedly evolving into diversified demanding fields with the immense collaboration of artificial intelligence, human psychology, chemical compounds, etc., with the medical domain infrastructure.

## Figures and Tables

**Table 1:** Parameters considered for system evaluation.

	Description
**Disease diagnosis**	Default value 10.0 is assigned to every system that helps diagnose diseases like anemia, arthritis, brain disorders, etc., related to the human body.
**Testing method**	Every system needs to follow one of the testing methods such as in vivo, in vitro, or both to perform disease diagnosis.
**Remote sensing**	Remote sensing of a system allows doctors to monitor patients’ health status by accessing information timely about their health status or their vital signs without the need for physical presence.
**Analysis type**	The system that is physically implemented or embedded in an application involved in disease diagnosis is considered real time. The system that involves the only simulation cannot be considered real time.
**Pain level**	The pain scale helps doctors decide accurate diagnosis and treatment plans to select medical devices for disease diagnosis.

**Table 2:** Parameter-based system evaluation.

System	Related To	Helps in Disease Diagnosis	Score	Testing Method	Score	Remote Sensing	Score	Real Time or Not Real Time	Score	Pain Level	Pain Scale	Total Score
Hemoglobin measurement [11]	Blood	Anemia	10	In vitro	6	No	5	Yes	10	Severe	7	78
Oxygen detection in blood [12]	Blood	Diabetes mellitus	10	In vitro	6	No	5	Yes	10	Moderate	6	76
Microfluidic cytometer [13]	Blood	Cardiovascular diseases	10	In vitro	6	No	5	Yes	10	Moderate	6	76
Finger powered agglutination lab chip [14]	Blood	Bacterial infection	10	In vitro	6	No	5	Yes	10	Moderate	5	74
Blood flow velocity detection [15]	Blood	Peripheral artery disease	10	In vivo	8	No	5	Yes	10	Severe	9	86
Glucose sensing [16]	Blood	Diabetes mellitus	10	both	10	Yes	10	Yes	10	Moderate	5	92
HIV diagnosis [17]	Blood	HIV	10	In vitro	6	Yes	10	Yes	10	Severe	7	88
Whole blood glucose analysis [18]	Blood	Diabetes mellitus	10	In vitro	6	No	5	Yes	10	Severe	7	78
Water salinity detection [19]	Blood	Diabetes mellitus	10	In vitro	6	Yes	10	Yes	10	None	0	74
Real-time toxic gas detection [20]	Blood	Leukemia	10	In vitro	6	Yes	10	Yes	10	Moderate	5	84
Portable chemical-free hemoglobin assay [21]	Blood	Anemia	10	In vitro	6	Yes	10	Yes	10	Moderate	6	86
Blood analysis [22]	Blood	Anemia	10	In vitro	6	Yes	10	Yes	10	Moderate	6	86
On-chip bioimaging [23]	Brain	Brain disorders	10	both	10	No	5	Yes	10	Severe	9	90
Neural activities measurement [24]	Brain	Brain disorders	10	In vivo	8	No	5	Yes	10	Severe	8	84
Intra brain image transmission [25]	Brain	Brain disorders	10	In vivo	8	Yes	10	Yes	10	Severe	9	96
Optogenetic device [26]	Brain	Brain disorders	10	In vivo	8	No	5	Yes	10	Severe	10	88
SiNAPS [27]	Brain	Brain disorders	10	In vivo	8	No	5	Yes	10	Severe	9	86
pH recording [28]	Brain	Brain disorders	10	In vitro	6	No	5	Yes	10	Severe	7	78
Positron imaging [29]	Brain	Brain disorders	10	In vivo	8	Yes	10	Yes	10	Severe	9	96
Active personal dosimeter [30]	Skin	Skin cancer	10	N/A	0	Yes	10	Yes	10	Mild	1	64
Disposable endoscopic application [31]	Intestines	GI tract diseases	10	In vivo	8	No	5	Yes	10	Severe	8	84
Wireless capsule endoscopy [32]	Intestines	GI tract diseases	10	In vivo	8	Yes	10	Yes	10	Severe	8	94
Endomicroscopic application [33]	Intestines	GI tract diseases	10	In vivo	8	No	5	No	5	Severe	8	74
IRIS application [34]	Eyes	N/A	0	N/A	0	Yes	10	No	5	None	0	32
Subretinal Implanted chip [35]	Eyes	Retinal diseases	10	Ex vivo	6	No	5	Yes	10	Severe	10	84
Visual Prosthesis [36]	Eyes	Retinal diseases	10	In vivo	8	No	5	Yes	10	Severe	10	88
Contactless pulse rate detection [37]	Heart	Shortness of breath	10	N/A	0	Yes	10	Yes	10	None	0	62
COVID-19 severity detection [38]	Lungs	COVID-19	10	In vitro	6	No	5	Yes	10	Severe	7	78
COVID-19 Cytokine storm monitoring [39]	Lungs	COVID-19	10	In vitro	6	No	5	Yes	10	Severe	7	78
COVID-19 risk assessment [40]	Lungs	COVID-19	10	In vitro	6	No	5	Yes	10	None	0	64
COVID-19 Saliva test [41]	Lungs	COVID-19	10	In vitro	6	No	5	Yes	10	None	0	64
Pose estimation platform for total hip arthroplasty [42]	Bones	Arthritis	10	In vivo	8	Yes	10	Yes	10	Severe	9	96
Knee Implants [43]	Bones	Arthritis	10	In vivo	8	Yes	10	Yes	10	Severe	9	96
Biofilm detection [44]	Bacteria Cells	GI tract diseases	10	In vivo	8	No	5	Yes	10	Severe	8	84
ePetri dish [45]	Bacteria Cells	Tissue damage	10	In vitro	6	Yes	10	Yes	10	Severe	7	88
DynAMITE [46]	Bacteria Cells	Breast cancer	10	N/A	0	No	5	Yes	10	Severe	9	70
Biomicrofluidic imaging [47]	Bacteria Cells	Cancer	10	In vitro	6	No	5	Yes	10	Severe	8	80
ELISA detector [48]	Bacteria Cells	Listeriosis	10	In vitro	6	No	5	Yes	10	Severe	7	78
FLIM [49]	Bacteria Cells	Hepatitis	10	In vitro	6	No	5	Yes	10	Severe	7	78
Quantifying protein dynamics [50]	Bacteria Cells	Cancer	10	both	10	No	5	Yes	10	Severe	9	90
Intracellular imaging and biosensing [51]	Bacteria Cells	Cancer	10	In vitro	6	No	5	Yes	10	Severe	7	78
Biochemiluminescence detection [52]	Bacteria Cells	Cholestatic liver disease	10	In vitro	6	No	5	Yes	10	Severe	7	78

**Table 3:** The utilization of complementary metal-oxide semiconductor image sensor CIS models in different medical devices performs disease diagnosis of vital signs, organs of the human body.

CMOS Technology/Image Sensor Model/Camera Module	Bacteria Cells	Blood	Bones	Brain	Eyes	Heart	Intestines	Lungs	Skin	Grand Total
**65 nm BSI CMOS**		1								1
**0.11 µm**	1									1
**0.15 µm**				1						1
**0.18 µm**	2	1	1	2	1		1			8
**0.35 µm**		2		4	1					7
**Apple iPhone Smartphone**	1									1
**Apple iPhone 5s Smartphone**	1									1
**Grasshopper 3 camera with Sony IMX174**								1		1
**LT225**	1									1
**MT9P031**	3	1								4
**Not mentioned**			1				2		1	4
**NAC Memrecam HX-5**								1		1
**NOON010PC30L**		2								2
**OV7680**					1					1
**OV8833**		1								1
**Samsung Galaxy S8 Smartphone**		1								1
**Samsung Galaxy SII Smartphone**		1								1
**Samsung S4 Smartphone**		1								1
**Samsung Galaxy S9 Smartphone**								1		1
**Sony Xperia E3 Smartphone**		1								1
**SONY α6100**								1		1
**TRDB_D5M**						1				1
**Grand Total**	9	12	2	7	3	1	3	4	1	42

## Data Availability

The data used in this review are from published primary studies, which are available in the public domain.

## Abbreviations

The following abbreviations are used in this manuscript:

CIS	CMOS Image Sensor
CMOS	Complementary Metal Oxide Semiconductor
CCD	Charge Coupled Device
WCE	Wireless Capsule Endoscopy
FPS	Frames Per Second
dB	Decibel
mm	Millimeter
µm	Micrometer
v/lux.s	Volts per luminance. Second
DR	Dynamic Range
SNR	Signal to Noise Ratio
BSI	Backside-illuminated

## Appendix A

**Table A1:** Parametric data of complementary metal-oxide semiconductor (CMOS) image sensors used in medical devices performing disease diagnosis.

S. No	Year	CMOS Technology/Camera Module	Pixel Size (µm)	Resolution	Pixel Pitch (µm)	Area	Power (W/mW)	SNR (dB)	Sensitivity (V/lux-s)	Frame Rate (fps)	Dynamic Range (dB)	Fill Factor (%)	Field	Application Name/Target
1	2014	0.35 µm	7.5 × 7.5	120 × 268	7.5	1048.6 µm × 2700 µm	N/A	N/A	N/A	58 Hz	N/A	44%	Implantable	Blood Flow Velocity Detection [15]
2	2014	0.35 µm	7.5 × 7.5	30 × 60	7.5	320 µm × 790 µm	N/A	N/A	N/A	10 fps	N/A	31%	Implantable	Glucose Sensors [16]
3	2011	MT9P031	2.2 × 2.2	2592 H × 1944 V	2.2	5.70 mm × 4.28 mm	381 mW	38.1 db	1.4	14 fps	70.1 db	N/A	Medical	Hemoglobin concentration measurement [11]
4	2011	0.18 µm	27 × 33	32 × 32	N/A	1.9 mm × 1.5 mm	625 µw	N/A	N/A	N/A	N/A	56%	Medical	Detection of luminescence response from a xerogel sensor array for O2 detection [12]
5	2017	65 nm BSI CMOS	1.1 × 1.1	1600 × 2056	1.1	1.69 mm × 2.24 mm	182.8 mW	N/A	1.05	45 fps	N/A	N/A	Medical	Microfluidic cytometer for complete blood count [13]
6	2019	OV8833	1.4 × 1.4	3264 × 2448	1.4	4.6 mm × 3.45 mm	291 mW	N/A	0.824	24 fps	67 dB	N/A	Medical	Finger powered agglutination lab chip [14]
7	2012	0.35 µm	15 × 7.5	128 × 268	N/A	2236 µm × 3171 µm	N/A	N/A	N/A	N/A	N/A	N/A	Biomedical	On-chip Bio Imaging sensor [23]
8	2012	0.35 µm	7.5 × 7.5	30 × 90 (needle), 120 × 268 (planar)	7.5	320 µm × 1025 µm (needle), 1000 µm × 3500 µm (planar)	N/A	N/A	N/A	N/A	N/A	N/A	Implantable	Monitoring Neural Activities [24]
9	2013	0.35 µm	7.5 × 7.5	60 × 60	7.5	1.0 mm × 1.0 mm	N/A	N/A	N/A	N/A	N/A	30%	Implantable	Wireless Imager for Intra Brain Image Transmission [25]
10	2017	0.35 µm	7.5 × 7.5	260 × 244	7.5	2200 × 2500	N/A	N/A	N/A	20 to 70 hz	N/A	N/A	Implantable	Optogenetic Device [26]
11	2018	0.18 µm	N/A	512 × 512	28	330 µm × 120 µm	N/A	N/A	N/A	N/A	N/A	N/A	Implantable	SiNAPS for Large Scale Neuro Recordings [27]
12	2019	0.15 µm	2.2 × 2.2	256 × 256	2	10.42 mm × 3.55 mm	N/A	N/A	N/A	3 0 fps	N/A	N/A	Implantable	Spatiotemporal pH Recording [28]
13	2018	0.18 µm	30 × 50	16 × 128	18	480 × 6400 µm	115 µW	N/A	N/A	N/A	N/A	N/A	Implantable	Positron Imaging in Rat Brain [29]
14	2013	N/A	5.6 × 5.6	640 × 480	5.6	11.43 mm × 11.43 mm	N/A	N/A	N/A	N/A	N/A	N/A	Medical	Active Personal Dosimeter [30]
15	2013	N/A	N/A	320 × 240	N/A	N/A	40 mw	53 dB	N/A	24 fps	N/A	25%	Biomedical	Wireless Capsule Endoscopy [32]
16	2012	0.18 µm	N/A	96 × 96	23	3 mm × 4 mm	6 µW	N/A	N/A	5 fps	N/A	N/A	Biomedical	Endomicroscope Applications [33]
17	2009	N/A	2.2 × 2.2	648 × 488	2.2	1.43 mm × 1.07 mm	80 mw	>36.5 db	1.1	30 fps	64 db	N/A	Medical	Disposable Endoscopic Applications [31]
18	2019	0.35 µm	114 × 117	64 × 64	N/A	4.3 mm × 3.2 mm	N/A	N/A	N/A	N/A	N/A	N/A	Biomedical	Stimulator for a sub retinal prosthesis [35]
19	2010	OV7680	2.2 × 2.2	640 × 480	2.2	1443.2 µm × 1082.4 µm	20 mW	N/A	0.56	30 fps	N/A	N/A	Implantable	Visual Prosthesis [36]
20	2018	0.18 µm	2.84 mm × 2.84 mm	174 × 144	2.84	1.72 mm × 1.65 mm	12.36 mw	N/A	N/A	520 fps	N/A	N/A	Medical	Iris detection for biometric applications [34]
21	2020	Grasshopper 3 camera with Sony IMX174	5.86 × 5.86	1920 × 1200	5.86	11.43 mm × 11.43 mm	N/A	N/A	10.45	48 fps	67.55 dB	N/A	Medical	COVID-19 severity detection [38]
22	2020	SONY α6100	N/A	6000 × 4000	N/A	23.5 mm × 15.6 mm	N/A	N/A	N/A	120 fps	N/A	N/A	Medical	COVID-19 Cytokine storm monitoring [39]
23	2020	NAC Memrecam HX-5	N/A	2560 × 1920	N/A	N/A	N/A	N/A	N/A	35 fps	N/A	N/A	Medical	COVID-19 risk assessment [40]
24	2021	Smartphone	N/A	N/A	N/A	N/A	N/A	N/A	N/A	N/A	N/A	N/A	Medical	COVID-19 Saliva test [41]
25	2016	N/A	N/A	240 × 240	N/A	N/A	N/A	N/A	N/A	8 fps	N/A	N/A	Implantable	Artificial Knee Implant Surgeries [43]
26	2019	0.18 µm, OV7660	15 × 15	200 × 200	15	3.5 mm × 3.5 mm	N/A	N/A	N/A	N/A	N/A	60%	Medical	Total Hip Arthroplasty Surgery [42]
27	2011	MT9P031	2.2 × 2.2	2592 × 1944	2.2	5.70 mm × 4.28 mm	N/A	38.1	1.4	14 fps	70.1 db	N/A	Biomedical	ePetri Dish [45]
28	2011	0.18 µm	50 × 50	2520 × 2560	50	12.8 cm × 12.8 cm	N/A	N/A	N/A	30 fps	65	N/A	Biomedical	DynAMITe (Dynamic range Adjustable for Medical Imaging Technology) for Bio-Medical Imaging [46]
29	2012	0.18 µm	10 × 10	128 × 128	10	2.5 mm × 5.0 mm	N/A	N/A	N/A	1750 fps	N/A	N/A	Biomedical	Bio-micro fluidic imaging system for cancer cell detection [47]
30	2014	MT9P031	2.2 × 2.2	2592 × 1944	2.2	5.70 mm × 4.28 mm	381 mw	38.1 db	1.4	14 fps	70.1 db	N/A	Biomedical	ELISA detector [48]
31	2017	0.11 µm	22.4 × 22.4	128 × 128	22.4	7.0 mm × 9.3 mm	N/A	N/A	N/A	45 fps	–	N/A	Biomedical	Real-Time Fluorescence Lifetime Imaging Microscopy [49]
32	2019	LT225	5.5 × 5.5	2048 × 1088	5.5	43 mm × 43 mm	N/A	N/A	N/A	170 fps	56.4	N/A	Biomedical	Quantifying Protein Dynamics [50]
33	2014	MT9P031	2.2 × 2.2	2592 × 1944	2.2	5.702 mm × 4.277 mm	381 mW	38.1 db	1.4	30 fps	70.1	N/A	Medical	Bio Film Detection [44]
34	2017	TRDB_D5M	2.2 × 2.2	2592 × 1944	2.2	5.702 mm × 4.277 mm	N/A	38.1 db	N/A	70 fps	70.1 db	N/A	Medical	Noncontact Heart rate detection [37]
